# Genetic diversity, phylogenetic structure and development of core collections in *Melilotus* accessions from a Chinese gene bank

**DOI:** 10.1038/s41598-019-49355-y

**Published:** 2019-09-10

**Authors:** Hongxiang Zhang, Rong Bai, Fan Wu, Wenli Guo, Zhuanzhuan Yan, Qi Yan, Yufei Zhang, Jinxing Ma, Jiyu Zhang

**Affiliations:** 1 State Key Laboratory of Grassland Agro-ecosystems; Key Laboratory of Grassland Livestock Industry Innovation, Ministry of Agriculture and Rural Affairs; College of Pastoral Agriculture Science and Technology, Lanzhou University, Lanzhou, 730020 P.R. China; 2 State Key Laboratory of Systematic and Evolutionary Botany, Institute of Botany, Chinese Academy of Sciences, Beijing, 100093 P.R. China; 3 National Quality Control & Inspection Centre for Grassland Industry Products, National Animal Husbandry Service, Ministry of Agriculture, Beijing, P.R. China

**Keywords:** Genetics, Plant sciences

## Abstract

*Melilotus* is an important forage legume, with high values as feed and medicine, and widely used as green manure, honey plant, and wildlife habitat enhancer. The genetic diversity, structure and subdivision of this forage crop remain unclear, and plant genetic resources are the basis of biodiversity and ecosystem diversity and have attracted increasing attention. In this study, the whole collection of 573 accessions from the National Gene Bank of Forage Germplasm (NGBFG, China) and 48 accessions from the National Plant Germplasm System (NPGS, USA) in genus *Melilotus* were measured with respect to five seed characters: seed length, width, width-to-length ratio, circumference and 100-seed weight. Shannon’ genetic diversity index (H’) and phenotypic differentiation (Pst) were calculated to better describe the genetic diversity. The ITS and *mat*K sequences were used to construct phylogenetic trees and study the genetic relationships within genus *Melilotu*. Based on seed morphology and molecular marker data, we preliminarily developed core collections and the sampling rates of *M*. *albus* and *M*. *officinalis* were determined to be 15% and 25%, respectively. The results obtained here provide preliminary sorting and supplemental information for the *Melilotus* collections in NGBFG, China, and establish a reference for further genetic breeding and other related projects.

## Introduction

*Melilotus* is a forage legume of family, including 19 annual and biennial species, and three of the species have been cultivated: *M*. *albus*, *M*. *officinalis*, and *M*. *indicus*^1–3^. In comparison with most other forages, *Melilotus* has the advantages of tolerating extreme environmental conditions and providing high seed yields^4,5^. The nitrogen fixation rate of *Melilotus* is superior to those of other legumes, and it is beneficial in crop rotations^6^. Additionally, *Melilotus* can be used as a crop fertilizer^7^ as well as nectar plants^8^ and has important medicinal value due to the biological activity of their coumarins, which have many biological and pharmacological activities, including anti-HIV and anti-tumor effects^9^. During the past few years, *Melilotus*, as a good leguminous forage, has received much attention^10,11^. Plant genetic resources are the most essential of the world’s natural resources and are of paramount importance for genetic improvement, germplasm innovation, and plant biology research; they play an important role in guaranteeing the food and nutrition security of an increasing population^12,13^. Abundant genetic resources have great potential to provide novel beneficial genes^14^.

During the last 3–4 decades, major advances have been made in conserving these resources^15,16^. Although a large number of plant germplasm materials have been conserved in gene banks, their use is limited because of their overwhelming amount and lack of management^17^. According to Food and Agriculture Organization (FAO) estimates, only 1 million to 2 million of the 7.4 million germplasm accessions are specific and non-repetitive, while the remaining germplasm accessions contain different levels of repetition. An assessment and classification of the diversity is essential for effective utilization of the germplasm, and core germplasm development has been proposed for better management and use of the collections available in gene banks^18,19^. A core collection can be defined as a minimum set of accessions representing maximum genetic diversity, and collections of the core set are described accurately and evaluated and managed carefully, for better conservation and utilization of germplasm accessions^20^. The common method of constructing a core set is to group the whole collection by morphological or molecular characteristics, then selecting the representative core accessions to form subcore groups and combining all subcore groups to construct the final core set^21,22^. The described core accessions could be more efficiently used for pre-breeding, genomic studies and conservation programs in gene banks.

Here, a total of 621 accessions of 18 *Melilotus* species, including the whole collection of 573 accessions from NGBFG, China, and 48 accessions from NPGS, USA, was analyzed to present a comprehensive view of the genetic diversity and phylogenetic structure among these accessions and provide the basis for constructing a core germplasm set. In our previous study, we selected 199 accessions to assess the genetic diversity in *Melilotus* and gain an initial understanding^23^. Seed morphology and the sequences of ITS and *mat*K were adopted to analyze genetic diversity and form core collections of *Melilotus*. Using seed traits to assess genetic diversity in the germplasm is advantageous in comparison with the use of other plant organs, as seeds are easy to collect and store^24^. More importantly, seed morphological traits can be utilized for species identification as well as selection criteria in crop improvement programs^25,26^. The nuclear DNA ITS and chloroplast DNA *mat*K have been widely applied in studies of inferring phylogenetic relationships at lower taxonomic levels and have been successfully used to analyze plant systematics^27–29^. The previous studies in Fabaceae indicated that the rate and pattern of ITS sequence mutation are appropriate for resolving relationships among species and genera^30^, as well as revealed that *mat*K sequence can be used in phylogenetic analyses to successfully resolve relationships even at the species level^31^. Additionally, these sequences showed high stability and discrimination in *Melilotus*^32^. Examining both sequences and seed morphology might be an efficient method to analyze variation among *Melilotus* accessions and construct core sets.

## Results

### Seed morphological characterization

**Figure 1:**
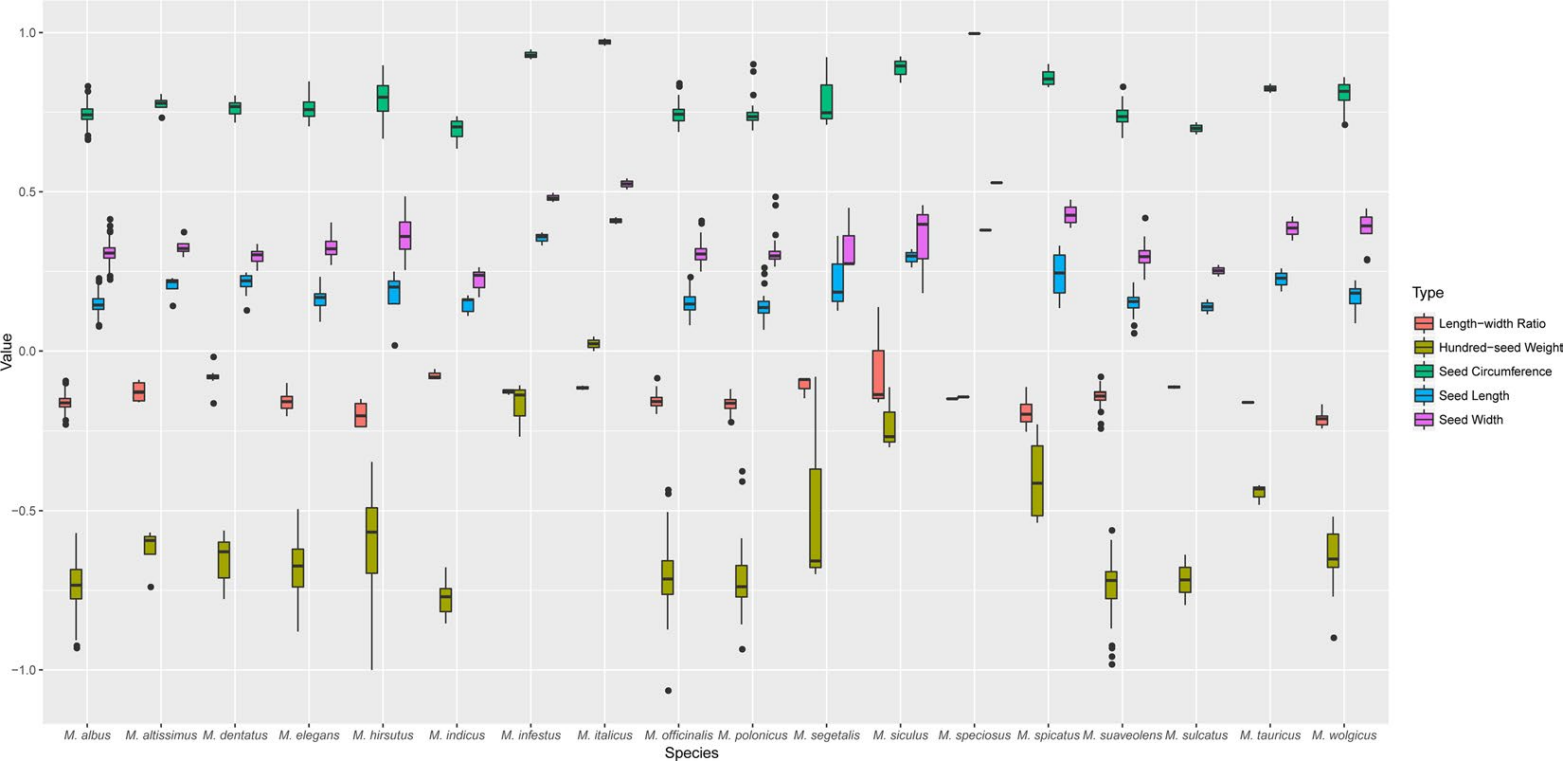
Morphologic variation analysis of five seed traits for 18 species. We calculated the logarithm of the values of five seed traits as ordinate in the box plot. Different traits are shown in different colors.

The morphologic traits in seeds are presented in Fig. 1 and Supplementary Table S1. The mean values of seed length, width, width-to-length ratio, circumference and 100-seed weight were 2.332 cm, 1.694 cm, 0.723, 6.564 cm and 0.365 g, respectively. According to Supplementary Table S1, an analysis of variance indicated significant (*p* < 0.05) differences among species, but the values of all traits overlapped a lot in range for many species (Fig. 1). The box plot revealed the relationships of seed size and shape of 18 species as well as indicated a small number of outliers. What’s more, we calculated the Pst parameter to assess the traits variation among species and the width-to-length ratio showed the lowest variation, while the 100-seed weight revealed the largest variation (the CV was 0.676 and the Pst parameter was 0.8473). The 100-seed weight and seed circumferences of *M*. *italicus*, *M*. *infestus*, *M*. *siculus* and *M*. *speciosus* were larger than those of the other species. Comparing the values of width-to-length ratio, circumference and 100-seed weight, the change tendencies of the latter two traits were similar since both two measures showed a positive correlation and reflected seed size. The width-to-length ratio was linked with the shape, and the difference among species was relatively small. Moreover, the CV values among species were larger than those within species, except for certain traits in a few species (the width-to-length ratios of *M*. *hirsutus* and *M*. *spicatus*, the circumference and 100-seed weight of *M*. *segetalis*).

### Cluster analysis

A total of 1145 sequences were newly amplified for this study. The nuclear DNA ITS sequences were successfully amplified for all 621 accessions, and the *mat*K sequences also performed well, with a high amplification rate of 99.3%. Based on these sequences, we constructed four phylogenetic trees to analyze the genetic diversity and phylogenetic structure of 18 species in *Melilotus*.

**Figure 2:**
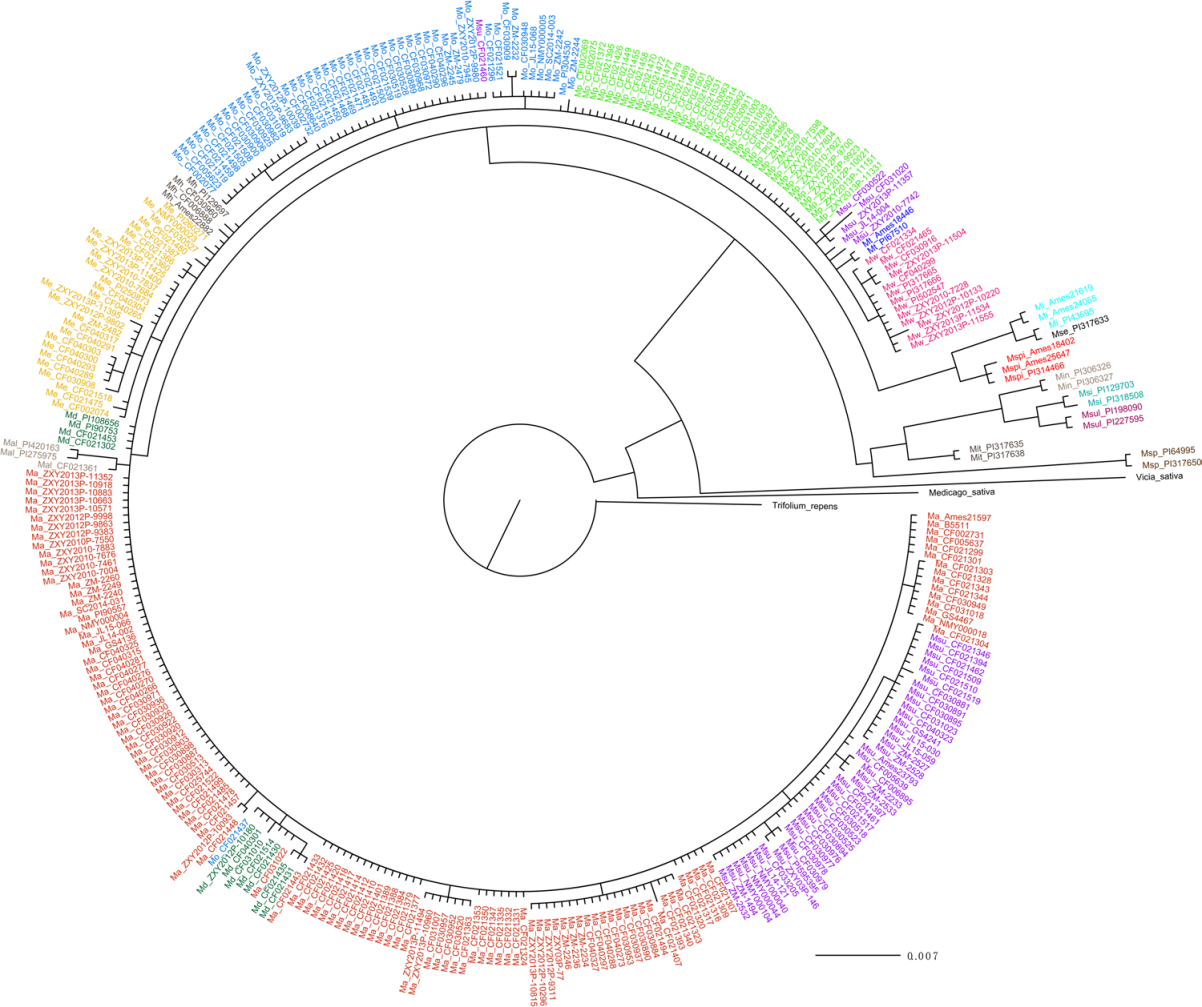
Bayesian tree of 18 species in *Melilotus* with branch lengths, based on ITS sequences. The abbreviations represent 18 species: Ma—*M*. *albus*, Mal—*M*. *altissimus*, Md—*M*. *dentatus*, Me—*M*. *elegans*, Mh—*M*. *hirsutus*, Mi—*M*. *indicus*, Min—*M*. *infestus*, Mit—*M*. *italicus*, Mo—*M*. *officinalis*, Mp—*M*. *polonicus*, Mse—*M*. *segetalis*, Msi—*M*. *siculus*, Ms—*M*. *speciosus*, Mpi—*M*. *spicatus*, Msu—*M*. *suaveolens*, Msul—*M*. *sulcatus*, Mt—*M*. *tauricus*, and Mw—*M*. *wolgicus*. See Supplement Table S3 for accession numbers.

A phylogenetic tree of 18 species based on ITS sequences is shown in Fig. 2, with *Vicia sativa*, *Medicago sativa* and *Trifolium repens* as outgroups. Most species showed distinct diversity, and the result was similar to that of the previous study, in which 18 species formed two groups^23^. Ten species, which were *M*. *albus*, *M*. *suaveolens*, *M*. *altissimus*, *M*. *dentatus*, *M*. *elegans*, *M*. *hirsutus*, *M*. *officinalis*, *M*. *polonicus*, *M*. *tauricus* and *M*. *wolgicus*, formed a clade as the first group, and the others formed the second group. Most species showed small intraspecific distances, and several species, including *M*. *albus*, *M*. *suaveolens* and *M*. *dentatus*, have a very close genetic relationship. Nevertheless, not all accessions of *M*. *suaveolens* gather in a subclade, since several accessions came together with *M*. *polonicus*. It might be caused by gene flow and the pervious study indicated *M*. *suaveolens* could successfully crossed with *M*. *albus* and *M*. *polonicus*^33^. In contrast, the *mat*K sequences didn’t perform well in assessing phylogenetic relationships in interspecific level. The diversity among 18 species revealed by *mat*K sequences was smaller, especially in the species *M*. *albus*, *M*. *altissimus*, *M*. *elegans*, *M*. *officinalis*, *M*. *polonicus*, *M*. *suaveolens* and *M*. *wolgicus*, as shown by their similar branch lengths (Supplementary Fig. S1), expect several accessions revealed variation with other accessions of the same species. The genetic diversity and relations could be reflected by the phylogenetic trees visually.

**Figure 3:**
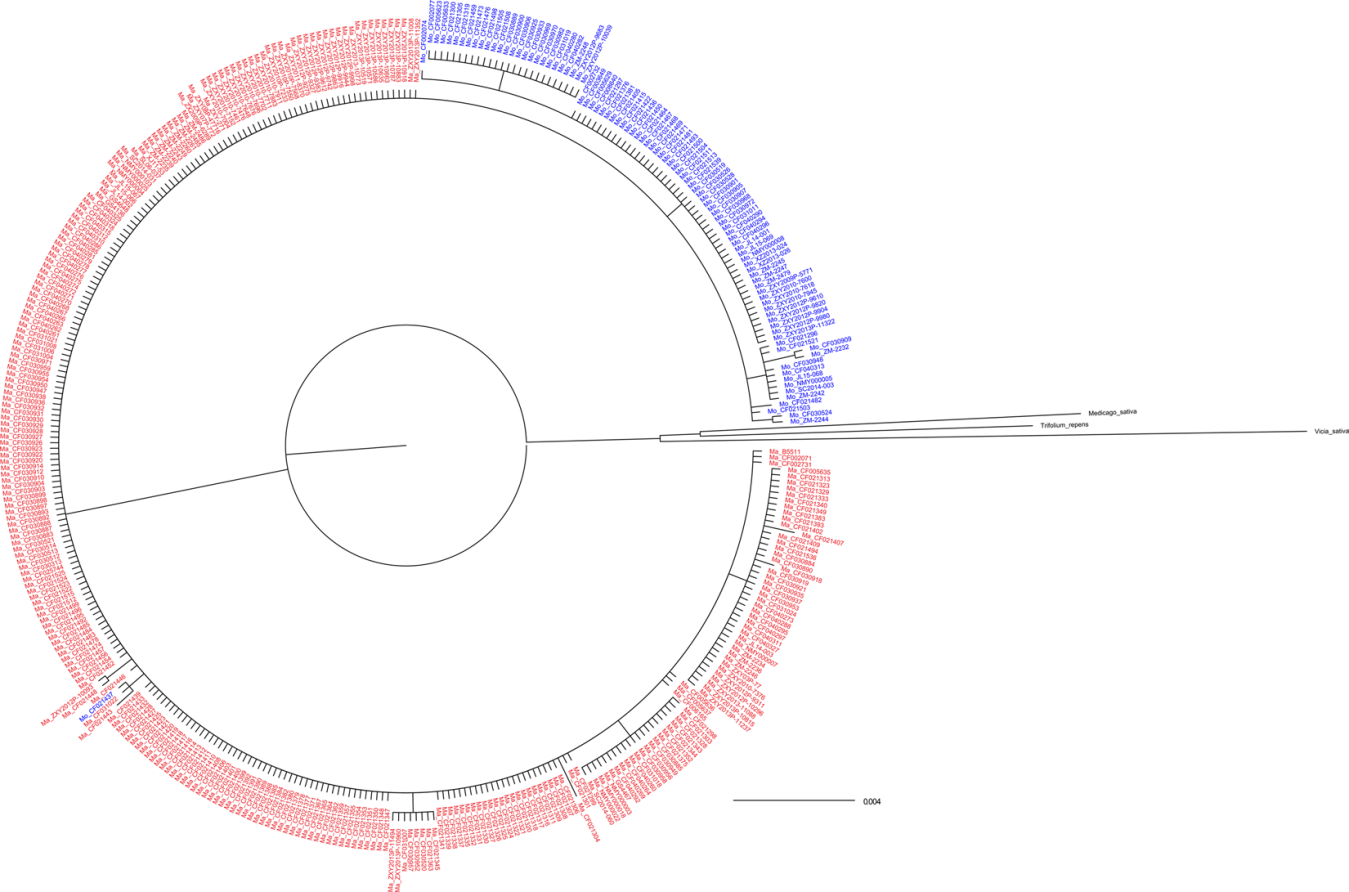
Bayesian tree of *M*. *albus* and *M*. *officinalis* with branch lengths, based on ITS sequences. Ma—*M*. *albus* and Mo—*M*. *officinalis*. See Supplement Table S3 for accession numbers.

Additionally, *M*. *albus* and *M*. *officinalis* have been widely cultivated, and both species have been studied many times^34^. We selected these two species to construct phylogenetic trees (Fig. 3 and Supplementary Fig. S2) to assess the genetic diversity exactly and create a reference for developing core collections. Nearly all accessions are divided by species, which provided additional evidence about *M*. *albus* and *M*. *officinalis* should be treated as genetically isolated taxa. Both two species have low intraspecific genetic diversity, and the trees that contained only these two species could reflect their diversity more effectively. Based on the ITS and *mat*K trees, the same species grouped together except several individual materials. The results showed small genetic distance within the species, and most accessions had the same branch lengths.

### Development of core collections

Two species, *M*. *albus* and *M*. *officinalis*, which stored large numbers of accessions in NGBFG, were selected to develop a representative core set. To determine an appropriate sampling ratio, six sampling proportions, 5%, 10%, 15%, 20%, 25% and 30%, were studied in our study. It is suggested that the coincidence rate (CR%) of range and the variable rate (VR%) for the coefficient of variation could evaluate the property of core collections^35^. We tried two different sampling methods, multiple clustering random sampling and multiple clustering preferred sampling. The core sets based on different sampling methods have different characteristics and are suitable for different studies. Random sampling can represent the genetic diversity structure of the initial collections and preferred sampling can keep the accessions with special or valuable characteristics in the initial collection^35^.

According to multiple clustering random sampling (Table 1), the values of CR% and VR% of *M*. *albus* did not change significantly as the sampling ratio reached 15%, and then genetic diversity of seed morphology declined smoothly as the sampling proportion increases. For *M*. *officinalis*, the proper sampling ratio was 25% or 20% based on the values of MD% and CR%, but the nucleotide diversity and haplotype diversity changed steadily until sampling proportion reached 25%. According to multiple clustering preferred sampling, nearly all MD% values are 0 and CR% values are 100%, and the VR% values changed steadily until sampling proportions of *M*. *albus* and *M*. *officinalis* reached 15% and 25%, respectively. However, through analysis of H’, nucleotide diversity and haplotype diversity, the variation of *M*. *officinalis* changed steadily from 20% sampling ratio. The core sets that have a good representativeness of the initial collection wouldn’t have rapid changes about diversity. To obtain more genetic diversity, the sampling ratios of *M*. *albus* and *M*. *officinalis* were determined to be 15% and 25%, respectively.

**Table 1:** Percentage of trait differences between the core collections and the initial collection at five sampling proportions.

Species	Sampling Methods	Sampling Ratio (%)	Evaluation Parameters			
			MD (%)	VD (%)	CR (%)	VR (%)
*M*. *albus*	Multiple clustering random sampling	5	0	100	97.6417	162.3625
		10	33.3333	66.6667	97.6417	138.2443
		15	0	100	98.2589	135.9037
		20	0	100	98.6975	132.2542
		25	0	100	98.6975	125.1218
		30	0	66.6667	98.6975	120.1121
	Multiple clustering preferred sampling	5	0	100	100	187.3465
		10	0	100	100	155.2074
		15	0	100	100	141.1182
		20	0	100	100	134.7276
		25	0	100	100	129.4654
		30	0	100	100	124.7173
*M*. *officinalis*	Multiple clustering random sampling	5	0	33.3333	72.3255	169.7614
		10	0	66.6667	94.6532	165.3490
		15	0	66.6667	94.6532	137.8036
		20	33.3333	33.3333	94.6532	125.1596
		25	0	0	96.2522	122.9678
		30	0	0	96.2522	118.0300
	Multiple clustering preferred sampling	5	33.3333	66.6667	90.9284	206.2261
		10	0	100	100	180.3112
		15	0	100	100	155.3382
		20	0	66.6667	100	146.3267
		25	0	66.6667	100	136.4903
		30	0	66.6667	100	133.0977

MD: percentage of significant difference (α = 0.05) between each core collection and the initial collection for means of traits, VD: percentage of significant difference (α = 0.05) between each core collection and the initial collection for variance of traits, CR%: coincidence rate, VR%: variable rate.

Overall, the coefficient of variation, genetic diversity index and sequence diversity were increased in the core collections, which was expected because diversity increased after the elimination of similar accessions during the development of the core germplasm sets. Additionally, the genetic diversity of *M*. *officinalis* is higher than that of *M*. *albus*, as shown in Table 2, and core collections were listed in Supplementary Table S2. The core collections, which maintained a high level of genetic diversity and were representative of the entire population, can be more efficiently used for breeding and phylogenetic studies than the whole collection.

**Table 2:** The comparison of the genetic diversity of the total collection *versus* the core sets.

Species	Sampling Methods	Sampling Ratio (%)	ITS		*mat*K		H’		
			Haplotype Diversity	Nucleotide Diversity	Haplotype Diversity	Nucleotide Diversity	Length-width Ratio	Seed Circumference	Hundred-seed Weight
*M*. *albus*	Multiple clustering random sampling	100	0.414	0.00085	0.547	0.00165	0.0066	0.0063	0.0058
		30	0.42	0.0007	0.68	0.00315	0.0100	0.0096	0.0094
		25	0.411	0.00068	0.631	0.00267	0.0107	0.0109	0.0101
		20	0.419	0.00069	0.62	0.00245	0.0117	0.0119	0.0114
		15	0.444	0.00082	0.684	0.00337	0.0127	0.0128	0.0129
		10	0.538	0.00105	0.664	0.00343	0.0140	0.0136	0.0096
		5	0.714	0.00156	0.705	0.0048	0.1562	0.1592	0.1645
	Multiple clustering preferred sampling	30	0.525	0.00183	0.558	0.00189	0.0106	0.0109	0.0108
		25	0.532	0.00196	0.566	0.00200	0.0111	0.0116	0.0115
		20	0.548	0.00217	0.581	0.00222	0.0120	0.0122	0.0120
		15	0.542	0.00247	0.577	0.00257	0.0127	0.0130	0.0131
		10	0.577	0.00157	0.591	0.00172	0.0141	0.0144	0.0140
		5	0.628	0.00226	0.562	0.00200	0.0160	0.0170	0.0156
*M*. *officinalis*	Multiple clustering random sampling	100	0.758	0.00234	0.736	0.00283	0.0104	0.0107	0.0109
		30	0.578	0.00126	0.808	0.00256	0.0145	0.0144	0.0146
		25	0.569	0.00122	0.769	0.00221	0.0146	0.0152	0.0146
		20	0.725	0.00197	0.772	0.00416	0.0153	0.0155	0.0162
		15	0.791	0.0023	0.813	0.00523	0.0164	0.0167	0.0154
		10	0.722	0.00206	0.833	0.00724	0.0179	0.0167	0.0172
		5	0.833	0.00181	0.833	0.00303	0.0178	0.0189	0.0189
	Multiple clustering preferred sampling	30	0.745	0.00380	0.775	0.00385	0.0143	0.0141	0.0135
		25	0.823	0.00440	0.830	0.00428	0.0145	0.0146	0.0140
		20	0.838	0.00515	0.850	0.00499	0.0151	0.0151	0.0148
		15	0.909	0.00348	0.910	0.00338	0.0159	0.0167	0.0160
		10	0.893	0.00350	0.893	0.00350	0.0172	0.0172	0.0174
		5	0.833	0.00350	0.833	0.00350	0.0178	0.0189	0.0178

H: genetic diversity index calculated using Shannon’s information index.

## Discussion

Conservation of plant genetic diversity is essential for present and future human well-being. Over the past few years, there have been many welcome developments in the conservation of forage germplasm resources^36^. As a high-quality forage species, *Melilotus* has many advantages and grows widely in China, and nearly 600 accessions of *Melilotus* were collected in NGBFG, China. In our previous study, we employed 199 accessions of 18 species to analyze genetic diversity^36^. The results indicated that *Melilotus* had high genetic variation among species, and thus, we further studied the genetic diversity and phylogenetic relationships of all *Melilotus* accessions in NGBFG, China. To better protect and utilize these resources, we analyzed the diversity of all accessions in NGBFG based on morphological and molecular data and developed core collections of two species. Morphological and molecular data can be analyzed separately or in combination to determine genetic diversity^37^. In addition, when constructing a core collection, a combination of both phenotypic and genotypic data is thought to be more useful than either one of these individually^38^. Based on seed morphological traits and the ITS and *mat*K sequences of *Melilotus*, we analyzed the genetic diversity of this genus and developed core sets to conserve and utilize germplasm resources efficiently.

According to Fig. 1 and Supplementary Table S1, the shape and size of seeds showed significant variation among and within species. Seed morphology in *Melilotus* showed a larger Pst parameter than some agronomic traits, such as plant height and dry matter yield^39^. These traits are important for seed establishment and survival^40^. Small-seeded species could produce more seeds for a given amount of energy than large-seeded species; however, large-seeded species, such as *M*. *italicus* and *M*. *speciosus*, develop seedlings that can better tolerate the many stresses encountered during establishment^41^. The variations in seed morphology could also reflect the wide range of habitats in *Melilotus*. This information on seed trait variation among accessions could also enhance cultivar development programs that focus on improving seedling survival or seed yield^42^. According to the phylogenetic trees based on the ITS sequences, almost all accessions could be divided by species. The first group, including *M*. *albus*, *M*. *suaveolens* was the recently diverged lineages, within the *Melilotus* genus. Additionally, the ITS sequences showed high discrimination in *Melilotus* in this study, while the results revealed that the *mat*K sequences did not perform as well as the ITS sequences. The *mat*K sequences might be more suitable for analyzing relationship at higher taxonomic levels^43^, but they can also reflect the variation among and within species to a certain degree^44^. Eighteen species included many subclades, but many accessions within each species showed the same branch lengths in both trees. Although the number of *M*. *albus* accessions was large, many repetitions were present, because of the frequent exchange of germplasm resources or resubmission of the same accessions. Clarifying the phylogenetic relationship and evaluating the genetic diversity of these accessions will provide a foundation for effective utilization of *Melilotus* accessions in NGBFG.

As the most widely-cultivated species in *Melilotus*, *M*. *albus* and *M*. *officinalis* are widely used in forage production and herbal medicine due to the biological activity of their coumarins^39^. Comparing *M*. *albus* with *M*. *officinalis*, the seed morphologies are similar (Fig. 1), and in fact, many taxonomic databases, including the USDA PLANTS database, the Integrated Taxonomic Information System, the BugwoodWiki website, and the Catalogue of Life website, have promulgated that the two species are merely conspecific colour morphs that do not merit taxonomic distinction or “accepts” *M*. *albus* both as a distinct species and as a subspecies of *M*. *officinalis* due to the similarity of morphological features and growing habits^45^. However, the phylogenetic trees we did in this study (Fig. 3 and Supplementary Fig. S2) with the previous studies^10,23^ indicated that *M*. *albus* and *M*. *officinalis* have a small genetic distance but are indeed distinct species. Furthermore, we developed core collections of these two species. Genetic parameters and cluster analysis were used to evaluate the efficiency of the development of the core germplasm set^46,47^. In this study, the genetic diversity index, haplotype diversity and nucleotide diversity of the core set were calculated and the core collections were evenly distributed across all clades in phylogenetic trees. Moreover, the sampling rates of *M*. *albus* and *M*. *officinalis* were different, which may be due to a difference in genetic variation. *Melilotus officinalis* showed higher diversity than *M*. *albus*, which might be caused by pollination type. *Melilotus albus* is cross-pollinating but self-fertile, while *M*. *officinalis* is self-incompatible^48^.

Core germplasm collections were constructed preliminarily, and additional studies (such as agronomic traits, plant morphology, biochemistry and other molecular marker data) are required to prefect the development of core germplasm collections. Although many rare alleles might not be captured in the core collections, developing core collections could help breeders increase efficiency and utilize genetic resources since cultivar development in *Melilotus* is still in the beginning stage. Besides, the results could also build a foundation for further physiological, genetic and molecular studies in *Melilotus* and provide a reference for future collection and conservation of *Melilotus* and other forages.

## Materials and Methods

### Plant materials

A total 621 accessions of *Melilotus* were evaluated in the study, and the details of these accessions are presented in Supplementary Table S3. The accessions in NGBFG, China, covered only nine species and most of the accessions belonged to five species, and thus, we added 48 accessions from NPGS, USA, that were studied in the previous study to analyze the phylogenetic structure and genetic diversity in *Melilotus*. To extract DNA, approximately 25 seeds of each accession were polished because of their hardness and then germinated at 24 °C after incubation in a 16-h light/8-h dark cycle. After two weeks, the seedlings were rinsed by distilled water, collected separately, frozen in liquid nitrogen and maintained at −80 °C until extracted.

### Seed morphology

Five characters of seeds were measured, including length, width, width-to-length ratio, circumference and 100-seed weight. We selected 100 seeds of each accession at random and measure their morphology using an analytical balance and WinSEEDLE, an image analysis system for morphological and disease analysis of seeds and needles.

### DNA extraction, amplification, and sequencing

Total genomic DNA was extracted from whole seedling material according to the SDS (sodium dodecyl sulfate) method^49^. The target DNA fragments, the internal transcribed spacer (ITS) and chloroplast locus *mat*K, were amplified and sequenced^50,51^. Amplification was performed by polymerase chain reactions (PCR) in 25-µL mixtures containing 12.25 µL of 2× reaction mix, 2 μL of each primer (1 μmol/mL), 2 μL of template genomic DNA (50 ng/μL), 0.25 µL of Golden DNA polymerase and 6.5 μL of deionized water. The primers and details of amplification programs were listed in Supplementary Table S4. Successful PCR products were sent to Shanghai Shenggong Biotechnological Ltd. (Shanghai, China) for sequencing.

### Alignment and diversity analysis

Both ends of the DNA sequences were trimmed to remove unalignable sequences upstream and downstream of the homologous sites by the Contig Express module of Vector NTI Suite 6.0 (InforMax, Inc) and aligned by DNAMAN 7.0^52,53^. The haplotype diversity and nucleotide diversity were computed by DnaSP 6.11^54^. The phylogenetic trees were drawn by ClustalW of MEGA 6.0 and MrBayes 3.2 software. The Bayesian method was adopted with the default settings and the GTR model with gamma-distributed rate variation across sites and a proportion of invariable sites (nst, 6; rates, invgamma)^55^ and operational generation number and sampling frequency were set to 100000000 and 100000, with *Medicago sativa*, *Trifolium repens* and *Vicia sativa* as outgroups. The morphological traits were analyzed using the statistical software package SPSS v16.0^37^. The coefficient of variation, phenotypic differentiation and Shannon’ genetic diversity index (H’) were calculated to analyze seed morphological diversity. The phenotypic differentiation coefficient (Pst) was calculated as follows: Pst = (σ^2^_t/s_)/(σ^2^_t/s_ + σ^2^_s_), where σ^2^_t/s_ is the variance portion among populations and σ^2^_s_ is the variance portion within populations^56^. Shannon’s diversity index was calculated as follows: H’ = − ∑ *pi* Ln *pi*, where *pi* is the proportion of each phenotypic trait^57^.

### Development of core collections

We used QGAStation 2.0, a software for classical quantitative genetics, to construct a core set according to the seed morphology. The strategy for constructing core collections adopted the least distance stepwise sampling based on genotypic values^58^, and Hu *et al*. (2000) suggested that standardized Euclidean distance combined with nearest distance method was an appropriate genetic distance for constructing core collections in this strategy^35^. We tried two sampling methods, multiple clustering random sampling and multiple clustering preferred sampling, to determine the appropriate sampling method and proportions^35^. Multiple clustering random sampling: one accession from each subgroup with two accessions at the lowest level of sorting is randomly selected. If there is only one accession in a subgroup, it is directly sampled for the next cluster. Multiple clustering preferred sampling: accessions with maximum or minimum values of traits are preferred to select from each subgroup at the lowest level of sorting. Both accessions are selected if two accessions in a subgroup have maximum or minimum values of the traits. The other procedures are similar to the random sampling strategy.

Six sampling proportions were chosen in the study, which were 5%, 10%, 15%, 20%, 25% and 30%. We calculated four parameters to evaluate the representation of the core germplasm at different sampling rates^58^: mean difference percentage (MD%), variance difference percentage (VD%), coincidence rate of range (CR%) and changeable rate of coefficient of variation (VR%). Additionally, the Shannon’ genetic diversity index of seed morphology and the haplotype diversity and nucleotide diversity of sequences were calculated to assess the genetic diversity of the core collections. According to the genetic diversity comparison of these core collections, we could determine the best sampling proportion, which was considered to be representative while maintaining a high level of genetic diversity.

## Supplementary information

Supplementary Information
