# Supplementary material for: Genetic diversity, phylogenetic structure and development of core collections in Melilotus accessions from a Chinese gene bank
Source: Sci Rep. 2019 Sep 10;9:13017. doi: 10.1038/s41598-019-49355-y (PMC6736865; doi:10.1038/s41598-019-49355-y)
Supplement: Supplementary file 1 — Supplementary Information [file 41598_2019_49355_MOESM1_ESM.pdf]

Genetic diversity, phylogenetic structure and development of core collections in *Melilotus* accessions from a Chinese gene bank

Hongxiang Zhang [1,2¶], Rong Bai [1¶], Fan Wu [1¶], Wenli Guo [1], Zhuanzhuan Yan [1], Qi Yan [1], Yufei Zhang [1], Jinxing Ma [3] and Jiyu Zhang [1*]

[1] State Key Laboratory of Grassland Agro-ecosystems; Key Laboratory of Grassland Livestock Industry Innovation, Ministry of Agriculture and Rural Affairs; College of Pastoral Agriculture Science and Technology, Lanzhou University; Lanzhou 730020, P. R. China.

[2] State Key Laboratory of Systematic and Evolutionary Botany, Institute of Botany, Chinese Academy of Sciences, Beijing 100093, P. R. China

[3] National Quality Control & Inspection Centre for Grassland Industry Products, National Animal Husbandry Service, Ministry of Agriculture, Beijing, P. R. China

[*]Author for correspondence:

Jiyu Zhang, E-mail address: zhangjy@lzu.edu.cn

[¶]These authors contributed equally to this work.

Species	Length-width Ratio*						Seed Circumference*						Hundred-seed Weight*					
	Mean	Range	SD	CV	H	Pst	Mean	Range	SD	CV	H	Pst	Mean	Range	SD	CV	H	Pst
M. albus	0.698	0.589-0.805	0.001	0.049			5.574	4.602-6.774	0.167	0.073			0.199	0.117-0.269	0.002	0.218		
M. altissimus	0.749	0.693-0.812	0.060	0.081			5.952	5.395-6.415	0.420	0.071			0.241	0.182-0.270	0.040	0.166		
M. dentatus	0.829	0.686-0.957	0.062	0.075			5.783	5.217-6.342	0.349	0.060			0.226	0.167-0.274	0.039	0.173		
M. elegans	0.697	0.626-0.793	0.041	0.059			5.773	5.074-7.024	0.439	0.076			0.212	0.132-0.320	0.044	0.207		
M. hirsutus	0.637	0.579-0.709	0.067	0.105			6.266	4.642-7.887	1.336	0.213			0.273	0.100-0.450	0.144	0.526		
M. indicus	0.838	0.816-0.878	0.025	0.030			4.971	4.319-5.457	0.432	0.087			0.170	0.140-0.210	0.025	0.149		
M. infestus	0.746	0.731-0.757	0.014	0.018			8.526	8.246-8.846	0.302	0.035			0.683	0.540-0.780	0.127	0.185		
M. italicus	0.776	0.773-0.778	0.004	0.005			9.343	9.094-9.592	0.352	0.038			1.055	1.000-1.110	0.078	0.074		
M. officinalis	0.692	0.637-0.821	0.001	0.048			5.543	4.877-6.920	0.109	0.059			0.188	0.086-0.367	0.001	0.162		
M. polonicus	0.681	0.605-0.759	0.036	0.053	1.860	0.564	5.560	4.926-7.941	0.587	0.106	1.831	0.761	0.198	0.116-0.420	0.059	0.295	1.380	0.847
M. segetalis	0.781	0.713-0.817	0.059	0.076			6.366	5.135-8.367	1.748	0.275			0.417	0.200-0.830	0.358	0.859		
M. siculus	0.726	0.719-0.732	0.007	0.009			7.740	6.959-8.410	0.732	0.095			0.603	0.500-0.770	0.146	0.242		
M. speciosus	0.710	0.710-0.710	-	-			9.926	9.926-9.926	-	-			0.720	0.720-0.720	-	-		
M. spicatus	0.651	0.560-0.771	0.089	0.137			7.253	6.735-7.964	0.547	0.075			0.418	0.290-0.590	0.143	0.343		
M. suaveolens	0.723	0.572-0.829	0.002	0.054			5.462	4.661-6.747	0.136	0.068			0.186	0.104-0.274	0.001	0.169		
M. sulcatus	0.771	0.761-0.780	0.013	0.017			5.007	4.785-5.228	0.313	0.063			0.195	0.160-0.230	0.049	0.254		
M. tauricus	0.692	0.687-0.696	0.005	0.007			6.680	6.464-6.910	0.223	0.033			0.360	0.330-0.360	0.026	0.073		
M. wolgicus	0.611	0.573-0.682	0.029	0.048			6.405	5.127-7.245	0.679	0.106			0.233	0.126-0.303	0.051	0.218		
Total	0.723	0.560-0.957	0.061	0.085			6.564	4.319-9.956	1.449	0.221			0.365	0.086-1.110	0.247	0.676		

Supplementary Table S1 The analysis of seed morphology of 18 species in *Melilotus*. *: significantly different at $p<0.05$, SD: standard deviation, CV: coefficient of variation, H': genetic diversity index calculated using Shannon's information index, Pst: phenotypic differentiation. The units of seed circumference and 100-seed weight are "cm" and "g", respectively.

Sampling methods	Species	Accessions						
Multiple clustering random sampling	*M. albus*	CF005636	CF021308	CF021311	CF021313	CF021317	CF021327	CF021329
		CF030884	CF030893	CF030897	CF030919	CF030923	CF030932	CF030952
		CF021379	CF021380	CF021382	CF021392	CF021414	CF021419	CF021443
		CF031021	CF031022	CF031024	CF040277	CF040281	CF040312	CF040325
		CF030512	JL15-066	NMY000004	NMY000018	NMY000103	XJ11-53	ZXY2010P-7568
		CF021371	CF031008	CF021536	CF021354	CF030957	CF021452	GS4136
		JL14-003	ZXY-271	ZXY2013P-10635				
	M. officinalis	CF002074	CF003649	CF005623	CF021300	CF021319	CF021422	CF021437
		CF030925	CF030933	CF030948	CF031011	CF031019	CF040282	JL14-001
		ZXY2010-7600	ZXY2010-7618	CF021473	CF021511	CF030528	NMY000008	XZ2013-026
		CF021468	JL15-069	ZM-2245				
Multiple clustering preferred sampling	*M. albus*	ZM-2239	ZXY2010-7004	ZXY2010P-7568	ZXY2011-8370	ZXY2012P-9998	ZXY2013P-10635	ZXY2013P-10663
		CF021452	CF021484	CF021536	CF030520	CF030884	CF030892	CF030893
		CF002071	CF021304	CF021313	CF021321	CF021324	CF021371	CF021379
		CF030947	CF030957	CF031021	CF031022	CF031024	CF040269	CF040277
		ZXY2013P-11494	JL14-002	SC2014-031	CF021443	CF021393	CF021423	CF021392

	CF030919	CF030920	CF030923	CF030931	GS4136	GS4648	CF040297
	CF021380	CF040292					
	CF002732	CF005623	CF021296	CF021381	CF021437	CF021450	CF021468
	CF021482	CF021504	CF030519	CF030925	CF030948	CF031011	CF031019
M. officinalis	CF040313	JL14-001	JL15-069	NMY000008	XZ2013-026	ZM-2479	ZXY2010-7618
	CF040282	ZXY2012P-9980	ZXY2013P-11322				

Supplementary Table S2 The accession numbers included in core collections.

Accessions	Species	Origin	Latitude	Longitude	Status	Seed Width	Seed Length	Seed Circumference	Hundred-seed Weight
Ames 21597	*M. albus*	Italy	12°34'	41°52'		1.976	1.379	5.158	0.160
B5511	*M. albus*					2.054	1.504	5.701	0.209
CF002071	*M. albus*				U	2.035	1.349	5.471	0.190
CF002731	*M. albus*	Canada	-75°25'	45°16'	C	2.010	1.403	5.51	0.191
CF005635	*M. albus*	Sichuan,China	104°11'	31°19'	U	2.022	1.388	5.458	0.184
CF005636	*M. albus*					2.069	1.407	5.615	0.180
CF005637	*M. albus*					2.225	1.506	6.004	0.219
CF006165	*M. albus*				U	2.056	1.434	5.611	0.189
CF021298	*M. albus*	Sichuan,China	105°3'	29°34'	U	2.230	1.4	5.848	0.195
CF021299	*M. albus*	Inner Mongoria,China	111°13'	39°52'	U	2.197	1.498	5.954	0.229
CF021301	*M. albus*	Sichuan,China	102°13'	31°55'	U	2.062	1.501	5.717	0.224
CF021303	*M. albus*	Shaanxi,China	109°22'	32°49'	U	1.983	1.358	5.366	0.170
CF021304	*M. albus*	Tibet,China	94°22'	29°40'	U	2.202	1.521	6.022	0.195
CF021306	*M. albus*	Sichuan,China	105°52'	32°39'	U	1.981	1.355	5.379	0.166
CF021307	*M. albus*	Shandong,China	118°21'	35°3'	U	2.179	1.475	5.894	0.199
CF021308	*M. albus*				U	2.146	1.492	5.83	0.211
CF021309	*M. albus*	Xinjiang,China	82°41'	37°3'	U	2.235	1.509	6.02	0.213
CF021311	*M. albus*	China	116°28'	39°48'	U	2.079	1.456	5.704	0.229
CF021313	*M. albus*				U	2.589	1.525	6.774	0.247
CF021316	*M. albus*				U	2.130	1.423	5.711	0.185
CF021317	*M. albus*				U	2.009	1.487	5.603	0.172
CF021318	*M. albus*				U	2.128	1.446	5.759	0.194
CF021320	*M. albus*				U	2.226	1.454	5.954	0.195

CF021321	*M. albus*				U	2.102	1.493	5.806	0.180
CF021322	*M. albus*				U	2.205	1.462	5.957	0.211
CF021323	*M. albus*				U	2.072	1.394	5.587	0.169
CF021324	*M. albus*				U	2.121	1.459	5.774	0.194
CF021325	*M. albus*				U	2.233	1.481	6.022	0.206
CF021326	*M. albus*				U	2.230	1.55	6.143	0.222
CF021327	*M. albus*				U	2.222	1.509	6.085	0.210
CF021328	*M. albus*				U	1.970	1.377	5.366	0.207
CF021329	*M. albus*				U	2.099	1.522	5.804	0.197
CF021330	*M. albus*				U	2.054	1.417	5.598	0.180
CF021331	*M. albus*				U	2.022	1.385	5.47	0.171
CF021332	*M. albus*				U	2.024	1.364	5.482	0.180
CF021333	*M. albus*				U	2.021	1.388	5.51	0.184
CF021335	*M. albus*				U	2.145	1.433	5.783	0.208
CF021337	*M. albus*				U	1.954	1.329	5.318	0.178
CF021338	*M. albus*				U	2.032	1.4	5.513	0.189
CF021339	*M. albus*				U	2.023	1.364	5.47	0.169
CF021340	*M. albus*				U	2.078	1.431	5.672	0.193
CF021341	*M. albus*				U	1.941	1.347	5.302	0.173
CF021343	*M. albus*				U	1.942	1.396	5.34	0.164
CF021344	*M. albus*				U	2.104	1.499	5.812	0.216
CF021345	*M. albus*				U	2.055	1.367	5.52	0.188
CF021347	*M. albus*	Russian Federation	37°34'	55°37'	U	1.868	1.358	5.15	0.173
CF021348	*M. albus*	Russian Federation	37°34'	55°37'	U	2.056	1.44	5.628	0.188
CF021349	*M. albus*	Russian Federation	37°34'	55°37'	U	2.046	1.367	5.486	0.178
CF021350	*M. albus*	Russian Federation	37°34'	55°37'	U	2.108	1.406	5.721	0.206

CF021351	*M. albus*	Russian Federation	37°34'	55°37'	U	2.005	1.39	5.487	0.189
CF021352	*M. albus*	Russian Federation	37°34'	55°37'	U	2.022	1.365	5.489	0.181
CF021353	*M. albus*	Russian Federation	37°34'	55°37'	U	2.100	1.367	5.625	0.191
CF021354	*M. albus*	Russian Federation	37°34'	55°37'	U	2.147	1.381	5.694	0.183
CF021355	*M. albus*	Russian Federation	37°34'	55°37'	U	1.921	1.267	5.174	0.162
CF021357	*M. albus*					1.958	1.41	5.415	0.193
CF021359	*M. albus*	Russian Federation	37°34'	55°37'	U	1.957	1.368	5.363	0.173
CF021362	*M. albus*	Russian Federation	37°34'	55°37'	U	2.021	1.46	5.599	0.189
CF021363	*M. albus*	Russian Federation	37°34'	55°37'	U	2.066	1.38	5.549	0.188
CF021364	*M. albus*	Russian Federation	37°34'	55°37'	U	2.112	1.352	5.644	0.187
CF021365	*M. albus*	Russian Federation	37°34'	55°37'	U	2.063	1.361	5.526	0.166
CF021367	*M. albus*					2.062	1.501	5.719	0.234
CF021371	*M. albus*	Beijing,China	116°28'	39°48'	C	2.190	1.417	5.843	0.201
CF021375	*M. albus*	Beijing,China	116°28'	39°48'	U	2.019	1.454	5.546	0.181
CF021377	*M. albus*	Russian Federation	72	58	U	1.965	1.35	5.339	0.162
CF021378	*M. albus*	Russian Federation	66	54	U	1.806	1.335	5.023	0.151
CF021379	*M. albus*	Russian Federation	40	60	U	1.941	1.33	5.554	0.165
CF021380	*M. albus*	Russian Federation	64	66	U	1.813	1.232	4.899	0.124
CF021382	*M. albus*	Russian Federation	62	59	U	1.833	1.281	5.02	0.161
CF021383	*M. albus*	Russian Federation	63	62	U	1.991	1.479	5.526	0.197
CF021384	*M. albus*	Russian Federation	54	54	U	1.969	1.321	5.316	0.149
CF021385	*M. albus*	Russian Federation	42	58	U	1.718	1.303	4.802	0.140
CF021388	*M. albus*	Russian Federation	63	57	U	1.897	1.376	5.258	0.166
CF021389	*M. albus*	Russian Federation	41	54	U	1.954	1.365	5.334	0.172
CF021390	*M. albus*	Russian Federation	74	70	U	1.875	1.37	5.179	0.176
CF021391	*M. albus*	Russian Federation	46	53	U	1.872	1.34	5.129	0.162

CF021392	*M. albus*	Russian Federation	68	52	U	1.676	1.228	4.644	0.119
CF021393	*M. albus*	Russian Federation	55	55	U	1.919	1.259	5.155	0.152
CF021399	*M. albus*				U	1.944	1.368	5.342	0.174
CF021400	*M. albus*				U	1.959	1.339	5.307	0.171
CF021401	*M. albus*				U	1.954	1.357	5.337	0.174
CF021402	*M. albus*				U	2.066	1.374	5.578	0.179
CF021403	*M. albus*				U	2.113	1.433	5.718	0.216
CF021406	*M. albus*				U	1.966	1.382	5.403	0.206
CF021407	*M. albus*				U	2.050	1.318	5.473	0.161
CF021408	*M. albus*				U	1.882	1.26	5.075	0.151
CF021409	*M. albus*				U	2.069	1.367	5.567	0.174
CF021410	*M. albus*				U	1.868	1.358	5.15	0.173
CF021411	*M. albus*				U	1.876	1.364	5.184	0.165
CF021412	*M. albus*				U	1.982	1.311	5.306	0.153
CF021413	*M. albus*				U	2.066	1.365	5.557	0.174
CF021414	*M. albus*				U	2.029	1.392	5.497	0.189
CF021416	*M. albus*				U	2.004	1.41	5.507	0.179
CF021417	*M. albus*				U	1.985	1.363	5.403	0.169
CF021418	*M. albus*				U	2.082	1.401	5.605	0.193
CF021419	*M. albus*				U	2.185	1.288	5.723	0.157
CF021420	*M. albus*				U	1.973	1.395	5.399	0.172
CF021421	*M. albus*				U	1.971	1.404	5.42	0.183
CF021423	*M. albus*				U	2.038	1.408	5.57	0.198
CF021425	*M. albus*				U	1.824	1.203	4.891	0.127
CF021427	*M. albus*				U	1.978	1.368	5.395	0.172
CF021428	*M. albus*				U	1.990	1.351	5.387	0.177

CF021432	*M. albus*				U	2.061	1.425	5.616	0.185
CF021433	*M. albus*	Russian Federation	37°34'	55°37'	U	2.022	1.41	5.492	0.178
CF021439	*M. albus*	Sichuan,China	105°51'	32°30'	U	1.955	1.344	5.339	0.190
CF021443	*M. albus*	Ningxia,China	107°07'	37°47'	U	2.079	1.664	6.051	0.240
CF021446	*M. albus*	China	116°28'	39°48'	U	1.908	1.386	5.302	0.195
CF021448	*M. albus*				U	2.175	1.347	5.817	0.195
CF021452	*M. albus*				U	1.832	1.474	5.277	0.175
CF021454	*M. albus*				U	1.946	1.338	5.295	0.162
CF021456	*M. albus*				U	1.981	1.302	5.336	0.166
CF021457	*M. albus*				U	1.993	1.39	5.421	0.197
CF021474	*M. albus*	Russian Federation	37°34'	55°37'	U	1.985	1.453	5.497	0.205
CF021478	*M. albus*	Russian Federation	37°34'	55°37'	U	2.082	1.401	5.605	0.197
CF021483	*M. albus*	Russian Federation	37°34'	55°37'	U	2.098	1.394	5.67	0.192
CF021484	*M. albus*	Russian Federation	37°34'	55°37'	U	1.674	1.193	4.602	0.126
CF021485	*M. albus*	Russian Federation	37°34'	55°37'	U	1.876	1.364	5.184	0.165
CF021492	*M. albus*	Beijing,China	116°28'	39°48'	U	1.940	1.463	5.452	0.215
CF021494	*M. albus*	Russian Federation	58	62	U	1.921	1.319	5.227	0.156
CF021495	*M. albus*	Russian Federation	67	53	U	1.921	1.37	5.281	0.178
CF021496	*M. albus*	Russian Federation	68	52	U	1.828	1.213	4.935	0.140
CF021499	*M. albus*	Russian Federation	77	72	U	1.995	1.391	5.441	0.184
CF021512	*M. albus*				U	2.030	1.389	5.502	0.177
CF021515	*M. albus*	Inner Mongoria,China	112°25'	40°39'	U	2.188	1.541	6.027	0.252
CF021522	*M. albus*				U	1.916	1.324	5.218	0.172
CF021523	*M. albus*	Inner Mongoria,China	119°19'	41°36'	U	2.243	1.544	6.09	0.242
CF021524	*M. albus*	Inner Mongoria,China	119°22'	43°58'	U	2.223	1.565	6.086	0.242
CF021525	*M. albus*	Inner Mongoria,China	108°31'	41°34'	U	2.207	1.466	5.922	0.225

CF021536	*M. albus*	Beijing,China	116°28'	39°48'	C	2.130	1.444	5.794	0.224
CF025744	*M. albus*	Xinjiang,China	82°45'	37°01'	U	2.195	1.484	5.92	0.234
CF030313	*M. albus*	Russian Federation	37°34'	55°37'	U	2.021	1.371	5.457	0.172
CF030512	*M. albus*	China	116°13'	40°10'	U	2.213	1.514	6.006	0.241
CF030513	*M. albus*	China	116°13'	40°10'	U	2.052	1.436	5.621	0.210
CF030514	*M. albus*	China	116°13'	40°10'	U	2.028	1.478	5.647	0.225
CF030520	*M. albus*	China	116°13'	40°10'	U	2.144	1.579	5.977	0.209
CF030521	*M. albus*	China	116°13'	40°10'	U	1.818	1.35	5.071	0.171
CF030883	*M. albus*	Jilin,China	126°25'	41°23'	U	1.963	1.527	5.551	0.213
CF030884	*M. albus*	Jilin,China	125°57'	40°59'	W	2.033	1.414	5.487	0.126
CF030885	*M. albus*					2.064	1.4	5.595	0.190
CF030887	*M. albus*	Britain	0°07'	51°30'	U	1.982	1.325	5.343	0.174
CF030888	*M. albus*	Israel	35°13'	31°47'	U	2.048	1.438	5.614	0.212
CF030890	*M. albus*	Australia	149°08'	35°17'	U	1.944	1.39	5.325	0.179
CF030892	*M. albus*	Jilin,China	126°25'	41°23'	U	1.972	1.369	5.359	0.161
CF030893	*M. albus*	Jilin,China	126°25'	41°23'	U	2.078	1.453	5.686	0.152
CF030897	*M. albus*	United States	-77°02'	38°54'	U	2.040	1.383	5.529	0.202
CF030898	*M. albus*	United States	-77°02'	38°54'	U	2.138	1.421	5.776	0.231
CF030899	*M. albus*	United States	-77°02'	38°54'	U	2.096	1.426	5.686	0.202
CF030903	*M. albus*	United States	-77°02'	38°54'	U	2.181	1.425	5.843	0.225
CF030904	*M. albus*	Canada	-75°25'	45°16'	U	1.984	1.345	5.359	0.186
CF030910	*M. albus*	Russian Federation	37°34'	55°37'	U	1.954	1.4	5.393	0.189
CF030912	*M. albus*	Russian Federation	37°34'	55°37'	U	1.856	1.366	5.15	0.166
CF030914	*M. albus*	Russian Federation	37°34'	55°37'	U	2.069	1.386	5.59	0.202
CF030918	*M. albus*	Russian Federation	37°34'	55°37'	U	2.056	1.338	5.474	0.175
CF030919	*M. albus*	Russian Federation	37°34'	55°37'	U	2.011	1.32	5.387	0.139

CF030920	*M. albus*	Russian Federation	37°34'	55°37'	U	1.873	1.386	5.248	0.184
CF030921	*M. albus*	Russian Federation	37°34'	55°37'	U	1.884	1.257	5.089	0.137
CF030922	*M. albus*	Russian Federation	37°34'	55°37'	U	1.939	1.244	5.185	0.142
CF030923	*M. albus*	Russian Federation	37°34'	55°37'	U	2.037	1.355	5.493	0.158
CF030926	*M. albus*	Altai Krai,Russian Federation			U	1.863	1.24	4.997	0.134
CF030927	*M. albus*	Russian Federation	37°34'	55°37'	U	1.894	1.28	5.095	0.151
CF030928	*M. albus*	Russian Federation	37°34'	55°37'	U	1.888	1.321	5.14	0.160
CF030929	*M. albus*	Kyrgyzstan	74°46'	42°53'	U	1.860	1.372	5.145	0.168
CF030930	*M. albus*	Romania	26°10'	44°23'	U	2.014	1.345	5.422	0.169
CF030931	*M. albus*	Bulgaria	23°12'	42°27'	U	1.859	1.391	5.193	0.159
CF030932	*M. albus*	Germany	13°02'	52°31'	U	2.006	1.421	5.5	0.184
CF030935	*M. albus*	Canada	-75°25'	45°16'	U	2.039	1.322	5.438	0.159
CF030936	*M. albus*	United States	-77°02'	38°54'	U	1.864	1.333	5.116	0.162
CF030937	*M. albus*	United States	-77°02'	38°54'	U	1.925	1.282	5.209	0.146
CF030938	*M. albus*	United States	-77°02'	38°54'	U	2.005	1.32	5.371	0.169
CF030947	*M. albus*	Sichuan,China	105°12'	32°34'	W	2.111	1.387	5.643	0.191
CF030949	*M. albus*	Sichuan,China	101°33'	30°02'	W	2.212	1.543	6.04	0.216
CF030950	*M. albus*	Sichuan,China	105°46'	31°51'	W	2.030	1.432	5.561	0.182
CF030952	*M. albus*	Beijing,China	115°44'	40°32'	W	2.017	1.471	5.583	0.215
CF030953	*M. albus*	Beijing,China	115°44'	40°32'	W	2.104	1.5	5.791	0.217
CF030954	*M. albus*	Hebei,China	114°35'	40°14'	W	1.925	1.523	5.487	0.224
CF030955	*M. albus*	Hebei,China	115°41'	40°57'	W	2.263	1.457	5.975	0.209
CF030956	*M. albus*	Hebei,China	115°49'	40°44'	W	2.255	1.549	6.102	0.250
CF030957	*M. albus*	Hebei,China	116°35'	39°35'	W	2.383	1.579	6.392	0.266
CF030958	*M. albus*	Hebei,China	115°43'	41°20'	W	2.086	1.548	5.782	0.239

CF030959	*M. albus*	Henan,China	32°06'	114°04'	W	2.104	1.413	5.641	0.191
CF030971	*M. albus*	Canada	-75°25'	45°16'	C	2.075	1.406	5.622	0.203
CF031004	*M. albus*	Russian Federation	37°34'	55°37'	U	1.966	1.355	5.341	0.173
CF031006	*M. albus*	Uzbekistan	69°13’	41°16’	U	2.282	1.542	6.188	0.245
CF031007	*M. albus*	Canada	-75°25'	45°16'	U	1.850	1.309	5.037	0.158
CF031008	*M. albus*	Canada	-75°25'	45°16'	U	1.911	1.326	5.235	0.164
CF031018	*M. albus*	Hebei,China	115°49'	40°49'	W	1.986	1.528	5.607	0.225
CF031021	*M. albus*	Hebei,China	115°43'	41°20'	W	1.982	1.513	5.614	0.230
CF031022	*M. albus*	Gansu,China	102°54'	35°05'	W	2.002	1.598	5.782	0.190
CF031024	*M. albus*				U	2.232	1.689	6.304	0.262
CF040260	*M. albus*	Inner Mongoria,China	116°50'	42°09'	W	2.217	1.473	5.961	0.243
CF040261	*M. albus*	China	116°28'	39°48'	W	1.970	1.391	5.422	0.182
CF040262	*M. albus*	China	116°28'	39°48'	W	2±0.0	1.357	5.375	0.186
CF040263	*M. albus*	China	116°28'	39°48'	W	2.060	1.439	5.612	0.196
CF040264	*M. albus*	China	116°28'	39°48'	W	2.041	1.44	5.591	0.202
CF040266	*M. albus*	China	116°28'	39°48'	W	2.183	1.514	5.923	0.206
CF040267	*M. albus*	China	116°28'	39°48'	W	2.189	1.474	5.919	0.224
CF040268	*M. albus*	China	116°28'	39°48'	W	2.212	1.456	5.93	0.202
CF040269	*M. albus*	China	116°28'	39°48'	W	2.247	1.364	5.856	0.173
CF040270	*M. albus*	Chita,Russian Federation	113°28'	52°3'	U	2.083	1.37	5.572	0.189
CF040271	*M. albus*	Chita,Russian Federation	113°28'	52°3'	U	2.074	1.32	5.508	0.163

CF040272	*M. albus*	Novosibirsk Oblast,Russian Federation	82°55'	55°04'	U	2.011	1.336	5.425	0.161
CF040273	*M. albus*	Novosibirsk Oblast,Russian Federation	82°55'	55°04'	U	2.045	1.364	5.483	0.153
CF040274	*M. albus*	Orenburg Oblast,Russian Federation	55°6'	51°46'	U	1.938	1.257	5.193	0.138
CF040275	*M. albus*	Yaroslavl Oblast,Russian Federation	39°52'	57°37'	U	1.932	1.31	5.24	0.149
CF040276	*M. albus*	Novosibirsk Oblast,Russian Federation			U	2.052	1.408	5.565	0.167
CF040277	*M. albus*	Novosibirsk Oblast,Russian Federation			U	1.999	1.262	5.3	0.139
CF040278	*M. albus*	Novosibirsk Oblast,Russian Federation			U	1.958	1.308	5.267	0.149
CF040279	*M. albus*	Rostov Oblast,Russian Federation	39°44'	47°8'	U	1.910	1.32	5.192	0.136
CF040281	*M. albus*	Kazakhstan	71°26'	51°11'	U	1.974	1.351	5.377	0.146
CF040285	*M. albus*					2.007	1.29	5.354	0.155

CF040286	*M. albus*					2.105	1.371	5.625	0.172
CF040288	*M. albus*	Karachayevo-Cherkesskaya			U	2.235	1.435	5.948	0.206
CF040292	*M. albus*	Kazakhstan	71°26'	51°11'	U	1.976	1.308	5.308	0.161
CF040295	*M. albus*	Ukraine	30°52'	50°45'	U	2.197	1.499	5.954	0.184
CF040297	*M. albus*	Chelyabinsk Oblast,Russian Federation	61°26'	55°9'	U	1.954	1.483	5.456	0.184
CF040310	*M. albus*	Shanxi,China	112°40'	40°02'	W	2.039	1.41	5.551	0.193
CF040311	*M. albus*	Shanxi,China	112°42'	40°10'	W	1.972	1.495	5.551	0.219
CF040312	*M. albus*	Shanxi,China	113°30'	40°01'	W	2.469	1.541	6.528	0.266
CF040315	*M. albus*	Beijing,China	115°53'	40°31'	W	1.972	1.423	5.479	0.202
CF040318	*M. albus*	Shanxi,China	112°31'	40°00'	W	2.173	1.51	5.936	0.218
CF040324	*M. albus*	Hebei,China	117°06'	41°29'	W	2.001	1.443	5.553	0.198
CF040325	*M. albus*	Inner Mongoria,China	118°37'	43°30'	W	2.314	1.609	6.314	0.250
CF040327	*M. albus*	Inner Mongoria,China	118°53'	42°32'	W	2.095	1.491	5.753	0.234
GS4136	*M. albus*					2.340	1.606	6.373	0.214
GS4467	*M. albus*					2.069	1.529	5.759	0.239
GS4648	*M. albus*					1.939	1.378	5.313	0.186
JL14-002	*M. albus*					2.175	1.542	5.998	0.229
JL14-003	*M. albus*					2.092	1.374	5.579	0.184
JL15-066	*M. albus*					1.922	1.407	5.338	0.178
JL15-067	*M. albus*					1.962	1.426	5.451	0.187
NMY000003	*M. albus*					2.222	1.516	6.014	0.225
NMY000004	*M. albus*					1.843	1.384	5.142	0.186

NMY000007	*M. albus*				2.064	1.455	5.657	0.219
NMY000018	*M. albus*				2.086	1.469	5.699	0.180
NMY000022	*M. albus*				2.019	1.394	5.49	0.195
NMY000025	*M. albus*				2.155	1.524	5.944	0.218
NMY000103	*M. albus*				2.159	1.591	5.986	0.255
PI 90557	*M. albus*	China, Manchuria	124°29'	45°19'	2.072	1.43	5.467	0.200
SC2014-031	*M. albus*				1.925	1.383	5.324	0.156
SC2014-060	*M. albus*				1.987	1.36	5.39	0.174
SL06-037	*M. albus*				2.117	1.492	5.785	0.180
XJ11-53	*M. albus*				1.971	1.404	5.445	0.202
ZM-2234	*M. albus*				2.086	1.507	5.78	0.239
ZM-2235	*M. albus*				2.119	1.54	5.875	0.257
ZM-2236	*M. albus*				2.112	1.496	5.795	0.233
ZM-2239	*M. albus*				2.331	1.598	6.36	0.269
ZM-2240	*M. albus*				2.189	1.456	5.862	0.232
ZM-2243	*M. albus*				2.161	1.534	5.944	0.248
ZM-2246	*M. albus*				2.136	1.598	5.981	0.240
ZM-2249	*M. albus*				2.039	1.441	5.58	0.207
ZM-2260	*M. albus*				2.140	1.439	5.806	0.206
ZM-2261	*M. albus*				2.146	1.494	5.851	0.210
ZM-2485	*M. albus*				2.139	1.434	5.767	0.211
ZM-2486	*M. albus*				2.150	1.647	6.078	0.219
ZX2009P-6289	*M. albus*				1.965	1.416	5.417	0.193
ZXY03P-77	*M. albus*				2.086	1.418	5.653	0.182
ZXY07P-772	*M. albus*				2.046	1.317	5.463	0.168
ZXY08P-47716	*M. albus*				1.958	1.372	5.353	0.173

ZXY2010-7004	*M. albus*	1.854	1.263	5.018	0.139
ZXY2010-7152	*M. albus*	1.884	1.341	5.203	0.161
ZXY2010-7376	*M. albus*	1.806	1.289	4.953	0.147
ZXY2010-7461	*M. albus*	2.001	1.361	5.383	0.165
ZXY2010-7476	*M. albus*	1.824	1.286	4.982	0.148
ZXY2010-7648	*M. albus*	1.918	1.329	5.199	0.143
ZXY2010-7676	*M. albus*	1.960	1.338	5.293	0.157
ZXY2010-7696	*M. albus*	2.118	1.371	5.612	0.174
ZXY2010-7702	*M. albus*	2.087	1.412	5.633	0.191
ZXY2010-7711	*M. albus*	1.879	1.305	5.117	0.153
ZXY2010-7883	*M. albus*	1.936	1.267	5.171	0.146
ZXY2010-7911	*M. albus*	1.898	1.29	5.168	0.138
ZXY2010P-7233	*M. albus*	2.047	1.341	5.479	0.171
ZXY2010P-7550	*M. albus*	2.075	1.373	5.567	0.175
ZXY2010P-7568	*M. albus*	2.006	1.294	5.324	0.169
ZXY2011-8370	*M. albus*	1.709	1.217	4.739	0.117
ZXY2012P-10093	*M. albus*	2.094	1.368	5.649	0.189
ZXY2012P-10296	*M. albus*	2.362	1.523	6.31	0.244
ZXY2012P-9275	*M. albus*	2.264	1.619	6.218	0.268
ZXY2012P-9311	*M. albus*	2.079	1.435	5.699	0.201
ZXY2012P-9327	*M. albus*	1.976	1.327	5.351	0.170
ZXY2012P-9383	*M. albus*	2.173	1.473	5.891	0.197
ZXY2012P-9612	*M. albus*	2.050	1.438	5.591	0.192
ZXY2012P-9742	*M. albus*	2.222	1.475	5.98	0.239

ZXY2012P-9863	*M. albus*	2.131	1.555	5.943	0.231
ZXY2012P-9916	*M. albus*	2.074	1.418	5.641	0.201
ZXY2012P-9944	*M. albus*	1.976	1.394	5.41	0.185
ZXY2012P-9998	*M. albus*	1.848	1.322	5.069	0.147
ZXY2013-10773	*M. albus*	2.137	1.458	5.806	0.216
ZXY2013-11088	*M. albus*	1.969	1.393	5.412	0.172
ZXY2013P-1051<br>9	*M. albus*	1.890	1.366	5.232	0.164
ZXY2013P-1057<br>1	*M. albus*	2.070	1.382	5.564	0.185
ZXY2013P-1058<br>6	*M. albus*	1.767	1.312	4.921	0.163
ZXY2013P-1063<br>5	*M. albus*	1.757	1.326	4.937	0.146
ZXY2013P-1066<br>3	*M. albus*	1.881	1.306	5.121	0.163
ZXY2013P-1078<br>7	*M. albus*	2.110	1.449	5.725	0.213
ZXY2013P-1081<br>5	*M. albus*	2.021	1.409	5.518	0.177
ZXY2013P-1088<br>3	*M. albus*	1.985	1.396	5.474	0.177
ZXY2013P-1091<br>8	*M. albus*	1.856	1.323	5.095	0.162
ZXY2013P-1096<br>0	*M. albus*	2.101	1.461	5.735	0.202

ZXY2013P-11008	*M. albus*					1.988	1.432	5.479	0.184
ZXY2013P-11237	*M. albus*					1.805	1.27	4.956	0.146
ZXY2013P-11352	*M. albus*					2.064	1.411	5.593	0.199
ZXY2013P-11494	*M. albus*					2.005	1.365	5.448	0.180
ZXY-271	*M. albus*					2.186	1.422	5.823	0.231
Ames 18376	*M. altissimus*	United States, Nebraska	-99°54'	41°29'		2.359	1.635	6.415	0.270
CF021361	*M. altissimus*	Russian Federation	37°34'	55°37'	U	1.972	1.384	5.395	0.182
PI 275975	*M. altissimus*	-	-	-		2.085	1.693	6.023	0.260
PI 420163	*M. altissimus*	France	-2°12'	46°13'		2.112	1.666	5.975	0.250
CF021302	*M. dentatus*	Chongqing,China	109°31'	31°3'	U	1.952	1.34	5.298	0.172
CF021430	*M. dentatus*					2.060	1.71	6.058	0.254
CF021431	*M. dentatus*				C	2.013	1.7	5.967	0.240
CF021435	*M. dentatus*	China	116°28'	39°48'	U	2.168	1.764	6.342	0.274
CF021453	*M.*				U	1.787	1.488	5.217	0.167

	dentatus								
CF021514	*M. dentatus*	Inner Mongoria,China	116°4'	43°57'	U	2.060	1.661	5.956	0.235
CF031010	*M. dentatus*	Jiangsu,China	120°15'	33°46'	W	2.047	1.744	6.096	0.268
CF040301	*M. dentatus*	Gansu,China	104°22'	33°46'	W	2.005	1.654	5.853	0.232
PI 108656	*M. dentatus*	Armenia	-45°2'	40°4'		1.814	1.736	5.561	0.250
PI 90753	*M. dentatus*	China	-104°11'	35°51'		1.942	1.614	5.723	0.220
ZXY2012P-10180	*M. dentatus*					1.885	1.573	5.546	0.170
CF002074	*M. elegans*	Canada	-75°25'	45°16'	C	1.937	1.311	5.239	0.170
CF021360	*M. elegans*	Russian Federation	37°34'	55°37'	U	2.040	1.463	5.63	0.197
CF021366	*M. elegans*	Russian Federation	37°34'	55°37'	U	2.192	1.483	5.98	0.222
CF021387	*M. elegans*	Russian Federation	60	60	U	1.864	1.276	5.074	0.158
CF021475	*M. elegans*	Russian Federation	37°34'	55°37'	U	2.089	1.473	5.728	0.210
CF021480	*M. elegans*	Russian Federation	37°34'	55°37'	U	2.093	1.379	5.628	0.201
CF021518	*M. elegans*				U	2.196	1.652	6.169	0.275
CF030527	*M. elegans*	China	116°13'	40°10'	U	2.276	1.611	6.293	0.246
CF030908	*M. elegans*	Shaanxi,China	108°02'	34°17'	W	1.924	1.338	5.255	0.166
CF040265	*M. elegans*				W	1.981	1.441	5.451	0.172
CF040289	*M. elegans*	Ukraine	30°52'	50°45'	U	2.269	1.474	6.08	0.262
CF040291	*M. elegans*	Kazakhstan	71°26'	51°11'	U	2.175	1.48	5.943	0.225
CF040293	*M. elegans*	Kazakhstan	71°26'	51°11'	U	2.262	1.587	6.207	0.260

CF040300	*M. elegans*	Ningxia,China	106°55'	37°18'	W	2.051	1.45	5.638	0.220
CF040303	*M. elegans*	Gansu,China	103°13'	34°3'	W	2.124	1.473	5.809	0.193
CF040304	*M. elegans*	Gansu,China	103°13'	34°3'	W	2.154	1.709	6.178	0.242
CF040317	*M. elegans*	Hebei,China	117°01'	42°27'	W	1.912	1.402	5.327	0.194
NMY000023	*M. elegans*					2.254	1.482	6.023	0.237
PI 250873	*M. elegans*	Iran	-53°41'	32°25'		2.222	1.451	5.869	0.220
PI 260271	*M. elegans*	Ethiopia, Shewa	-37°48'	9°9'		2.532	1.689	7.024	0.320
ZM-2482	*M. elegans*					2.110	1.367	5.637	0.221
ZXY2010-7684	*M. elegans*					2.038	1.332	5.459	0.152
ZXY2010-7837	*M. elegans*					1.973	1.236	5.192	0.132
ZXY2012P-9802	*M. elegans*					2.078	1.479	5.701	0.198
ZXY2013P-11395	*M. elegans*					2.084	1.542	5.77	0.212
ZXY2013P-11400	*M. elegans*					2.281	1.662	6.313	0.262
ZXY2013P-11425	*M. elegans*					1.867	1.45	5.25	0.147
Ames 22882	*M. hirsutus*	Russian Federation	105°19'	61°31'		1.796	1.04	4.642	0.100
CF006888	*M. hirsutus*	China	116°28'	39°48'	U	2.387	1.62	6.484	0.289
CF030960	*M. hirsutus*	Inner Mongoria,China	112°04'	42°01'	W	2.195	1.556	6.052	0.254
PI 129697	*M. hirsutus*	Sweden	-18°38'	60°7'		3.057	1.776	7.887	0.450
Ames 21619	*M. indicus*	United States, Nebraska	-99°54'	41°29'		1.763	1.444	5.034	0.180

Ames 24055	*M. indicus*	Egypt	30°48'	26°49'		1.696	1.455	5.328	0.180
PI 107562	*M. indicus*	Uzbekistan	64°35'	41°22'		1.476	1.296	4.319	0.140
PI 260756	*M. indicus*	Turkey	35°14'	38°57'		1.546	1.289	4.613	0.160
PI 308524	*M. indicus*	Peru	-75°0'	-9°11'		1.832	1.495	5.457	0.150
PI 43595	*M. indicus*	-	-	-		1.769	1.454	5.077	0.210
PI 306326	*M. infestus*	Algeria	2°29'	27°13'		2.936	2.146	8.246	0.540
PI 306327	*M. infestus*	Italy	12°34'	41°52'		3.018	2.285	8.485	0.730
PI 306328	*M. infestus*	Hungary	19°30'	47°9'		3.137	2.356	8.846	0.780
PI 317635	*M. italicus*	Czechoslovakia	121°2'	14°28'		3.483	2.629	9.592	1.110
PI 317638	*M. italicus*	Israel	34°51'	31°2'		3.215	2.501	9.094	1.000
CF002074	*M. officinalis*	Beijing,China	116°28'	39°48'	C	2.083	1.53	5.862	0.253
CF002077	*M. officinalis*	Beijing,China	116°28'	39°48'	C	1.940	1.391	5.339	0.205
CF002732	*M. officinalis*	Canada	-75°25'	45°16'	C	1.885	1.313	5.137	0.151
CF003649	*M. officinalis*	Gansu,China	100°10'	39°7'	U	2.048	1.465	5.643	0.215
CF005623	*M. officinalis*	Gansu,China	103°49'	36°4'	C	2.042	1.445	5.637	0.180
CF005629	*M. officinalis*	Gansu,China	103°49'	36°4'	C	2.107	1.457	5.743	0.209
CF005633	*M. officinalis*	Beijing,China	116°28'	39°48'	C	2.032	1.452	5.604	0.229
CF008640	*M. officinalis*	Inner Mongoria,China	111°39'	40°49'	U	1.848	1.306	5.066	0.176

CF021296	*M. officinalis*				U	2.295	1.555	6.241	0.220
CF021297	*M. officinalis*	Canada	-75°25'	45°16'	U	2.078	1.421	5.664	0.181
CF021300	*M. officinalis*				U	1.986	1.382	5.412	0.174
CF021305	*M. officinalis*	Gansu,China	102°37'	37°55'	U	2.080	1.425	5.659	0.211
CF021319	*M. officinalis*				U	2.252	1.441	5.967	0.186
CF021376	*M. officinalis*	Russian Federation	64	66	U	1.904	1.303	5.132	0.143
CF021381	*M. officinalis*	Russian Federation	37°34'	55°37'	U	1.777	1.242	4.877	0.148
CF021405	*M. officinalis*				U	1.993	1.37	5.429	0.175
CF021415	*M. officinalis*				U	1.911	1.324	5.21	0.168
CF021422	*M. officinalis*				U	1.983	1.33	5.336	0.180
CF021436	*M. officinalis*	Inner Mongoria,China	111°13'	39°52'	C	2.035	1.403	5.548	0.194
CF021437	*M. officinalis*	Gansu,China	102°52'	37°28'	U	2.076	1.704	6.033	0.233
CF021450	*M. officinalis*				U	2.077	1.354	5.531	0.170

CF021459	*M. officinalis*	Sichuan,China	101°51'	26°42'	U	2.080	1.405	5.628	0.188
CF021464	*M. officinalis*	Russian Federation	37°34'	55°37'	U	1.871	1.35	5.147	0.162
CF021467	*M. officinalis*	Russian Federation	37°34'	55°37'	U	1.998	1.42	5.505	0.198
CF021468	*M. officinalis*	Russian Federation	37°34'	55°37'	U	1.942	1.332	5.265	0.177
CF021469	*M. officinalis*	Russian Federation	37°34'	55°37'	U	1.913	1.291	5.153	0.155
CF021471	*M. officinalis*	Russian Federation	37°34'	55°37'	U	2.132	1.406	5.709	0.188
CF021473	*M. officinalis*	Russian Federation	37°34'	55°37'	U	2.079	1.387	5.615	0.172
CF021476	*M. officinalis*	Russian Federation	37°34'	55°37'	U	1.991	1.338	5.342	0.184
CF021481	*M. officinalis*	Russian Federation	37°34'	55°37'	U	2.051	1.384	5.544	0.201
CF021482	*M. officinalis*	Russian Federation	37°34'	55°37'	U	1.867	1.205	4.975	0.148
CF021493	*M. officinalis*	Russian Federation	58	56	U	1.921	1.254	5.143	0.145
CF021498	*M. officinalis*	Russian Federation	66	54	U	2.023	1.337	5.417	0.182
CF021500	*M. officinalis*	Russian Federation	74	66	U	1.966	1.302	5.29	0.157

CF021503	*M. officinalis*	Russian Federation	49	61	U	2.078	1.399	5.609	0.178
CF021504	*M. officinalis*	Russian Federation	36	80	U	2.512	1.598	6.763	0.357
CF021505	*M. officinalis*	Russian Federation	42	78	U	2.072	1.391	5.591	0.167
CF021508	*M. officinalis*	Henan,China	32°13'	114°26'	W	1.865	1.414	5.246	0.186
CF021511	*M. officinalis*				U	1.844	1.302	5.062	0.174
CF021513	*M. officinalis*	Gansu,China	107°37'	35°4'	U	1.926	1.35	5.259	0.160
CF021521	*M. officinalis*	Inner Mongoria,China	120°4'	43°52'	U	2.198	1.491	5.943	0.251
CF021539	*M. officinalis*	Jilin,China	126°40'	43°38'	U	1.842	1.304	5.051	0.168
CF030519	*M. officinalis*	China	116°13'	40°10'	U	2.049	1.45	5.622	0.230
CF030524	*M. officinalis*	China	116°13'	40°10'	U	1.870	1.357	5.23	0.201
CF030526	*M. officinalis*	China	116°13'	40°10'	U	1.951	1.377	5.353	0.193
CF030528	*M. officinalis*	China	116°13'	40°10'	U	1.889	1.382	5.297	0.211
CF030889	*M. officinalis*	Australia	149°08'	35°17'	U	2.064	1.395	5.579	0.208

CF030896	*M. officinalis*	China	116°28'	39°48'	U	1.912	1.325	5.211	0.168
CF030900	*M. officinalis*	United States	-77°02'	38°54'	U	2.207	1.468	5.946	0.218
CF030901	*M. officinalis*	United States	-77°02'	38°54'	U	2.027	1.411	5.52	0.209
CF030905	*M. officinalis*	Canada	-75°25'	45°16'	U	1.935	1.381	5.377	0.193
CF030906	*M. officinalis*	Canada	-75°25'	45°16'	U	1.981	1.379	5.4	0.191
CF030907	*M. officinalis*	China	116°28'	39°48'	U	1.881	1.313	5.171	0.173
CF030909	*M. officinalis*	Russian Federation	37°34'	55°37'	U	1.893	1.315	5.122	0.147
CF030925	*M. officinalis*	Russian Federation	37°34'	55°37'	U	1.990	1.375	5.433	0.086
CF030933	*M. officinalis*	Belgium	4°21'	50°51'	U	1.960	1.408	5.392	0.181
CF030948	*M. officinalis*	Sichuan,China	102°17'	27°55'	W	2.014	1.468	5.553	0.197
CF030968	*M. officinalis*				U	1.970	1.383	5.403	0.195
CF030969	*M. officinalis*	Hebei,China	114°44'	39°14'	W	2.144	1.65	6.059	0.247
CF030970	*M. officinalis*	Canada	-75°25'	45°16'	C	1.902	1.417	5.313	0.194

CF030972	*M. officinalis*	Canada	-75°25'	45°16'	C	2.098	1.454	5.7	0.213
CF030982	*M. officinalis*	Hebei,China	115°46'	41°42'	W	1.941	1.476	5.458	0.220
CF031011	*M. officinalis*	Sichuan,China	103°36'	32°38'	W	2.221	1.576	6.102	0.196
CF031019	*M. officinalis*	Hebei,China	115°47'	40°55'	W	1.907	1.405	5.319	0.173
CF040280	*M. officinalis*	Kazakhstan	71°26'	51°11'	U	2.357	1.546	6.37	0.272
CF040282	*M. officinalis*	Kazakhstan	71°26'	51°11'	U	2.512	1.655	6.792	0.313
CF040290	*M. officinalis*	Kazakhstan	71°26'	51°11'	U	2.016	1.381	5.467	0.182
CF040294	*M. officinalis*	Kazakhstan	71°26'	51°11'	U	2.140	1.393	5.736	0.190
CF040296	*M. officinalis*	Czech Republic	14°25'	50°05'	U	1.850	1.225	4.953	0.134
CF040313	*M. officinalis*	Shanxi,China	112°27'	40°01'	W	2.115	1.562	5.891	0.238
JL14-001	*M. officinalis*					2.182	1.527	5.985	0.233
JL15-068	*M. officinalis*					2.240	1.564	6.141	0.257
JL15-069	*M. officinalis*					2.308	1.613	6.322	0.270

NMY000005	*M. officinalis*				2.217	1.498	6.002	0.206
NMY000008	*M. officinalis*				2.094	1.431	5.675	0.210
PI 304530	*M. officinalis*	Turkey	-35°14'	38°57'	2.017	1.299	5.269	0.160
SC2014-003	*M. officinalis*				2.135	1.576	5.944	0.235
XZ2013-024	*M. officinalis*				2.110	1.636	6.012	0.242
XZ2013-026	*M. officinalis*				2.199	1.664	6.202	0.273
ZM-2232	*M. officinalis*				2.223	1.597	6.133	0.243
ZM-2242	*M. officinalis*				1.982	1.495	5.559	0.221
ZM-2244	*M. officinalis*				2.052	1.516	5.733	0.230
ZM-2245	*M. officinalis*				1.992	1.518	5.627	0.237
ZM-2247	*M. officinalis*				2.025	1.479	5.62	0.230
ZM-2248	*M. officinalis*				2.066	1.502	5.764	0.253
ZM-2479	*M. officinalis*				2.322	1.481	6.152	0.210

ZXY2010-7600	*M. officinalis*					1.931	1.28	5.187	0.141
ZXY2010-7618	*M. officinalis*					1.827	1.245	4.934	0.148
ZXY2010-7945	*M. officinalis*					1.991	1.359	5.427	0.173
ZXY2012P-10039	*M. officinalis*					1.943	1.362	5.322	0.182
ZXY2012P-9610	*M. officinalis*					1.937	1.337	5.256	0.169
ZXY2012P-9683	*M. officinalis*					2.009	1.475	5.589	0.197
ZXY2012P-9820	*M. officinalis*					2.009	1.36	5.429	0.165
ZXY2012P-9904	*M. officinalis*					2.054	1.479	5.704	0.230
ZXY2012P-9980	*M. officinalis*					1.849	1.326	5.063	0.158
ZXY2013P-11322	*M. officinalis*					2.560	1.699	6.92	0.367
CF002069	*M. polonicus*	Beijing,China	116°28'	39°48'	U	1.977	1.434	5.5	0.213
CF002075	*M. polonicus*	Beijing,China	116°28'	39°48'	U	2.129	1.479	5.825	0.236
CF021372	*M. polonicus*	Beijing,China	116°28'	39°48'	C	2.105	1.433	5.701	0.259

CF021395	*M. polonicus*	Russian Federation	49	59	U	1.989	1.372	5.415	0.175
CF021426	*M. polonicus*				U	1.982	1.365	5.399	0.179
CF021449	*M. polonicus*				U	1.989	1.279	5.305	0.172
CF021455	*M. polonicus*				U	1.918	1.339	5.222	0.160
CF021458	*M. polonicus*				U	2.222	1.392	5.899	0.212
CF021470	*M. polonicus*	Russian Federation	37°34'	55°37'	U	1.937	1.27	5.179	0.160
CF021472	*M. polonicus*	Russian Federation	37°34'	55°37'	U	1.875	1.286	5.084	0.161
CF021477	*M. polonicus*	Russian Federation	37°34'	55°37'	U	1.982	1.353	5.386	0.171
CF021479	*M. polonicus*	Russian Federation	37°34'	55°37'	U	1.987	1.348	5.4	0.180
CF021489	*M. polonicus*	Beijing,China	116°28'	39°48'	C	2.025	1.451	5.594	0.215
CF021497	*M. polonicus*	Russian Federation	46	54	U	1.963	1.36	5.363	0.180
CF021501	*M. polonicus*	Russian Federation	60	70	U	1.878	1.261	5.061	0.145
CF021502	*M. polonicus*	Russian Federation	40	57	U	2.059	1.308	5.473	0.183

CF021531	*M. polonicus*					2.008	1.429	5.512	0.223
CF030063	*M. polonicus*				U	1.979	1.352	5.375	0.172
CF030314	*M. polonicus*	Russian Federation	37°34'	55°37'	U	2.000	1.323	5.387	0.171
CF030517	*M. polonicus*	China	116°13'	40°10'	U	2.311	1.628	6.358	0.257
CF030902	*M. polonicus*	United States	-77°02'	38°54'	U	1.938	1.399	5.371	0.191
CF030911	*M. polonicus*	Russian Federation	37°34'	55°37'	U	1.915	1.373	5.282	0.219
CF031003	*M. polonicus*	Russian Federation	37°34'	55°37'	U	1.962	1.49	5.512	0.204
CF031005	*M. polonicus*	Armenia	44°32'	40°10'	U	2.040	1.358	5.475	0.195
CF040287	*M. polonicus*	Stavropol Krai,Russian Federation			U	2.142	1.314	5.619	0.156
PI 108647	*M. polonicus*	Former Soviet Union	120°60'	24°47'		2.865	1.745	7.539	0.390
PI 314386	*M. polonicus*	Former Soviet Union	120°60'	24°47'		3.045	1.824	7.941	0.420
ZM -2529	*M. polonicus*					2.046	1.472	5.614	0.218
ZM-2478	*M.*					1.929	1.366	5.301	0.169

	polonicus							
ZXY2010-7598	*M. polonicus*				1.848	1.271	5.006	0.139
ZXY2010-7794	*M. polonicus*				1.841	1.27	5.016	0.150
ZXY2010-7804	*M. polonicus*				1.882	1.166	4.926	0.116
ZXY2010-7927	*M. polonicus*				1.968	1.276	5.285	0.147
ZXY2012P-10021	*M. polonicus*				2.002	1.415	5.503	0.197
ZXY2012P-9700	*M. polonicus*				2.020	1.478	5.609	0.209
ZXY2012P-9923	*M. polonicus*				2.093	1.401	5.64	0.201
ZXY2013P-11151	*M. polonicus*				2.045	1.409	5.562	0.182
ZXY2013P-11331	*M. polonicus*				2.069	1.423	5.659	0.208
PI 317633	*M. segetalis*	Algeria	2°29'	27°13'	1.88	1.53	5.597	0.220
PI 317649	*M. segetalis*	Czechoslovakia	121°2'	14°28'	2.814	2.299	8.367	0.830
PI 43597	*M. segetalis*	-	-	-	1.878	1.339	5.135	0.200
PI 129703	*M. siculus*	Malta	14°22'	35°56'	1.52	2.088	8.41	0.770

PI 318508	*M. siculus*	Greece	21°49'	39°4'		2.868	1.986	7.852	0.500
PI 33366	*M. siculus*	Former Soviet Union	120°59'	24°46'		2.5	1.83	6.959	0.540
PI 317650	*M. speciosus*	Canada, Manitoba	-98°48'	53°45'		3.375	2.396	9.926	0.720
Ames 18402	*M. spicatus*	United States, Nebraska	-99°54'	41°29'		2.563	1.579	6.925	0.310
Ames 25647	*M. spicatus*	Ukraine, Krym	34°6'	44°57'		2.438	1.365	6.735	0.290
PI 314466	*M. spicatus*	Uzbekistan	64°35'	41°22'		2.986	1.956	7.386	0.480
PI 317644	*M. spicatus*	Algeria	2°29'	27°13'		2.777	2.141	7.964	0.590
Ames 18444	*M. suaveolens*	United States, Nebraska	-99°54'	41°29'		1.714	1.136	4.745	0.110
Ames 23793	*M. suaveolens*	Mongolia	103°50'	46°51'		1.92	1.135	5.009	0.160
CF005639	*M. suaveolens*	Hubei,China	114°18'	30°36'	U	1.690	1.2	4.661	0.104
CF006895	*M. suaveolens*	China	116°28'	39°48'	U	1.912	1.387	5.284	0.202
CF021314	*M. suaveolens*				U	2.612	1.494	6.747	0.206
CF021346	*M. suaveolens*	Gansu,China	101°48'	36°29'	U	2.022	1.473	5.589	0.216
CF021394	*M. suaveolens*	Russian Federation	60	63	U	1.690	1.295	4.793	0.135

CF021397	*M. suaveolens*	Hebei,China	110°46'	31°42'	U	1.904	1.421	5.334	0.156
CF021440	*M. suaveolens*	Gansu,China	102°56'	34°24'	U	2.055	1.628	5.869	0.241
CF021441	*M. suaveolens*	Ningxia,China	107°07'	37°47'	U	2.076	1.468	5.701	0.174
CF021442	*M. suaveolens*	Gansu,China	103°23'	34°40'	U	1.997	1.405	5.483	0.140
CF021445	*M. suaveolens*	China	116°28'	39°48'	U	1.948	1.43	5.4	0.193
CF021447	*M. suaveolens*	Gansu,China	103°23'	34°40'	U	2.061	1.601	5.854	0.208
CF021460	*M. suaveolens*				U	2.086	1.443	5.687	0.192
CF021461	*M. suaveolens*	Inner Mongoria,China	117°48'	43°12'	U	2.158	1.491	5.888	0.219
CF021462	*M. suaveolens*	Shanxi,China	112°44'	37°40'	U	1.978	1.454	5.489	0.202
CF021463	*M. suaveolens*	Beijing,China	116°28'	39°48'	U	1.892	1.346	5.174	0.160
CF021466	*M. suaveolens*	Russian Federation	37°34'	55°37'	U	1.939	1.346	5.298	0.155
CF021506	*M. suaveolens*	Russian Federation	77	63	U	1.953	1.363	5.373	0.155
CF021507	*M. suaveolens*	Russian Federation	66	66	U	1.948	1.354	5.316	0.157

CF021509	*M. suaveolens*	Sichuan,China	102°33'	32°48'	U	2.164	1.499	5.841	0.191
CF021510	*M. suaveolens*	Sichuan,China	102°58'	32°4'	U	2.159	1.606	6.025	0.231
CF021517	*M. suaveolens*				U	1.758	1.356	4.942	0.168
CF021519	*M. suaveolens*	China	116°28'	39°48'	U	1.850	1.343	5.187	0.158
CF021520	*M. suaveolens*	Jilin,China	123°31'	44°36'	U	1.987	1.451	5.528	0.191
CF021526	*M. suaveolens*	Gansu,China	99°38'	38°48'	U	2.043	1.455	5.599	0.184
CF021527	*M. suaveolens*	Inner Mongoria,China	117°28'	43°14'	U	2.083	1.464	5.702	0.206
CF021532	*M. suaveolens*	Gansu,China	102°56'	35°02'	U	2.002	1.606	5.833	0.227
CF021537	*M. suaveolens*	Hubei,China	110°23'	31°42'	U	1.940	1.355	5.284	0.146
CF021540	*M. suaveolens*	Jilin,China	126°45'	43°35'	U	1.990	1.438	5.475	0.191
CF025745	*M. suaveolens*	Jilin,China	126°40'	43°38'	U	1.839	1.372	5.135	0.172
CF030515	*M. suaveolens*	China	116°13'	40°10'	U	1.834	1.403	5.185	0.186
CF030516	*M. suaveolens*	China	116°13'	40°10'	U	2.013	1.399	5.503	0.184

CF030518	*M. suaveolens*	China	116°13'	40°10'	U	1.901	1.454	5.379	0.211
CF030522	*M. suaveolens*	China	116°13'	40°10'	U	1.973	1.458	5.526	0.211
CF030523	*M. suaveolens*	China	116°13'	40°10'	U	2.001	1.516	5.638	0.219
CF030525	*M. suaveolens*	China	116°13'	40°10'	U	2.115	1.494	5.804	0.206
CF030881	*M. suaveolens*	Gansu,China	103°30'	34°34'	W	1.957	1.438	5.43	0.197
CF030891	*M. suaveolens*	Gansu,China	103°30'	34°34'	W	1.988	1.403	5.422	0.137
CF030894	*M. suaveolens*	Inner Mongoria,China	130°12'	44°39'	W	2.004	1.496	5.645	0.196
CF030895	*M. suaveolens*	Jilin,China	125°57'	40°59'	W	2.003	1.432	5.524	0.171
CF030946	*M. suaveolens*	Hubei,China	110°36'	31°42'	W	1.979	1.366	5.409	0.119
CF030964	*M. suaveolens*	Jilin,China	126°33'	43°49'	W	1.955	1.344	5.29	0.171
CF030973	*M. suaveolens*	Hebei,China	116°02'	41°34'	W	1.881	1.433	5.303	0.191
CF030974	*M. suaveolens*	Hebei,China	115°25'	40°00'	W	2.013	1.419	5.514	0.197
CF030975	*M. suaveolens*	Hebei,China	115°42'	40°38'	W	1.927	1.395	5.306	0.183

CF030976	*M. suaveolens*	Hebei,China	114°47'	39°51'	W	1.816	1.417	5.169	0.193
CF030977	*M. suaveolens*	Hebei,China	114°39'	39°42'	W	2.100	1.508	5.779	0.221
CF030978	*M. suaveolens*	Hebei,China	115°49'	40°55'	W	2.053	1.491	5.677	0.214
CF030979	*M. suaveolens*	Beijing,China	115°29'	39°47'	W	1.915	1.396	5.332	0.187
CF030981	*M. suaveolens*	Beijing,China	116°44'	40°42'	W	1.674	1.281	4.737	0.150
CF030983	*M. suaveolens*	Hebei,China	114°53'	39°52'	W	2.083	1.516	5.79	0.215
CF031012	*M. suaveolens*	Sichuan,China	102°17'	27°55'	W	1.755	1.351	4.955	0.154
CF031013	*M. suaveolens*	Sichuan,China	102°57'	33°34'	W	2.140	1.643	6.089	0.248
CF031015	*M. suaveolens*	Hebei,China	115°28'	39°55'	W	1.883	1.445	5.33	0.198
CF031016	*M. suaveolens*	Hebei,China	115°45'	41°07'	W	1.917	1.409	5.327	0.181
CF031020	*M. suaveolens*	Hebei,China	115°49'	40°49'	W	2.136	1.405	5.704	0.201
CF031023	*M. suaveolens*	Gansu,China	102°45'	35°18'	W	1.949	1.463	5.446	0.172
CF033205	*M. suaveolens*	Fujian,China	118°43'	26°22'	W	1.790	1.314	4.956	0.148

CF040306	*M. suaveolens*	Hubei,China	110°40'	31°45'	W	1.927	1.379	5.3	0.117
CF040307	*M. suaveolens*	Inner Mongoria,China	124°39'	45°57'	W	1.820	1.423	5.185	0.178
CF040308	*M. suaveolens*	Beijing,China	115°51'	40°08'	W	1.993	1.475	5.59	0.208
CF040309	*M. suaveolens*	Hebei,China	114°42'	39°14'	W	1.874	1.441	5.287	0.192
CF040314	*M. suaveolens*	Hebei,China	117°50'	41°48'	W	1.923	1.316	5.232	0.162
CF040316	*M. suaveolens*	Beijing,China	115°53'	40°32'	W	1.891	1.36	5.229	0.178
CF040319	*M. suaveolens*	Inner Mongoria,China	118°36'	43°15'	W	1.844	1.383	5.155	0.181
CF040320	*M. suaveolens*	Hebei,China	117°12'	41°16'	W	1.985	1.444	5.488	0.202
CF040321	*M. suaveolens*	Hebei,China	117°46'	41°58'	W	1.835	1.366	5.15	0.169
CF040322	*M. suaveolens*	Shanxi,China	113°29'	38°57'	W	2.216	1.612	6.143	0.244
CF040323	*M. suaveolens*	Inner Mongoria,China	118°47'	42°20'	W	1.988	1.436	5.491	0.199
CF040326	*M. suaveolens*	Inner Mongoria,China	118°21'	43°03'	W	1.942	1.423	5.395	0.192
GS4241	*M. suaveolens*					2.052	1.534	5.696	0.189

JL14-004	*M. suaveolens*				2.023	1.339	5.448	0.175
JL14-121	*M. suaveolens*				1.719	1.425	5.005	0.187
JL15-030	*M. suaveolens*				2.070	1.424	5.657	0.171
JL15-059	*M. suaveolens*				1.919	1.384	5.3	0.167
NMY000040	*M. suaveolens*				1.966	1.46	5.491	0.188
NMY000044	*M. suaveolens*				2.222	1.585	6.107	0.256
NMY000104	*M. suaveolens*				2.051	1.465	5.659	0.193
PI 593408	*M. suaveolens*	United States, South Dakota	-99°54'	43°58'	1.802	1.337	4.952	0.150
PI 595395	*M. suaveolens*	United States, Iowa	-93°5'	41°52'	1.714	1.38	4.854	0.180
ZM-1494	*M. suaveolens*				1.991	1.442	5.506	0.192
ZM-2233	*M. suaveolens*				2.171	1.548	5.998	0.236
ZM-2237	*M. suaveolens*				2.159	1.461	5.833	0.229
ZM-2241	*M. suaveolens*				2.289	1.599	6.313	0.274

ZM-2477	*M. suaveolens*	1.914	1.357	5.253	0.169
ZM-2480	*M. suaveolens*	1.945	1.375	5.343	0.190
ZM-2481	*M. suaveolens*	1.822	1.334	5.072	0.155
ZM-2483	*M. suaveolens*	1.722	1.258	4.794	0.141
ZM-2484	*M. suaveolens*	1.730	1.288	4.819	0.156
ZM-2527	*M. suaveolens*	2.144	1.527	5.898	0.229
ZM-2528	*M. suaveolens*	2.007	1.469	5.566	0.197
ZM-2530	*M. suaveolens*	2.205	1.479	5.964	0.203
ZM-2531	*M. suaveolens*	2.157	1.495	5.857	0.201
ZM-2532	*M. suaveolens*	2.154	1.478	5.882	0.203
ZM-2533	*M. suaveolens*	2.126	1.455	5.767	0.204
ZXY03P-146	*M. suaveolens*	1.892	1.34	5.185	0.148
ZXY2010-7742	*M. suaveolens*	2.166	1.397	5.764	0.193

ZXY2013P-11357	*M. suaveolens*					2.071	1.493	5.741	0.218
PI 198090	*M. sulcatus*	Morocco	-7°5'	31°47'		1.863	1.453	5.228	0.230
PI 227595	*M. sulcatus*	Tunisia	-9°32'	33°53'		1.714	1.304	4.785	0.160
Ames 18446	*M. tauricus*	United States, Nebraska	-99°54'	41°29'		2.223	1.538	6.464	0.330
Ames 25789	*M. tauricus*	Ukraine, Krym	34°6'	44°57'		2.432	1.693	6.667	0.370
PI 67510	*M. tauricus*	Ukraine, Krym	34°6'	44°57'		2.645	1.817	6.91	0.380
CF021334	*M. wolgicus*				U	1.927	1.315	5.218	0.170
CF021465	*M. wolgicus*	Russian Federation	37°34'	55°37'	U	2.533	1.56	6.639	0.260
CF030916	*M. wolgicus*	Russian Federation	37°34'	55°37'	U	2.331	1.409	6.11	0.219
CF040299	*M. wolgicus*	Germany	13°02'	52°31'	U	2.778	1.617	7.245	0.267
PI 317665	*M. wolgicus*	Denmark	9°30'	56°15'		2.396	1.373	6.131	0.210
PI 317666	*M. wolgicus*	Czechoslovakia	121°2'	14°28'		2.437	1.538	6.179	0.200
PI 502547	*M. wolgicus*	Russian Federation	105°19'	61°31'		2.337	1.465	6.157	0.220

ZXY2010-7228	*M. wolgicus*	1.938	1.223	5.127	0.126
ZXY2012P-10133	*M. wolgicus*	2.633	1.52	6.803	0.265
ZXY2012P-10220	*M. wolgicus*	2.470	1.518	6.538	0.223
ZXY2013P-11504	*M. wolgicus*	2.668	1.666	7.019	0.297
ZXY2013P-11534	*M. wolgicus*	2.620	1.568	6.86	0.272
ZXY2013P-11555	*M. wolgicus*	2.801	1.649	7.239	0.303

Supplementary Table S3 Information on 621 accessions included in the study. The units of seed width, length and circumference are “cm”, and the unit of 100-seed weight is “g”. The abbreviations in Status are as follows: W, wild; C, cultivar; S, strain; U, unverified.

Region	Primer squence (5'-3')	Annealing temperature (°C)	Extension time (s)	Aligned lengths/bp
*mat*K	F:CCCRTYCATCTGGAAATCTTGGTTC<br>R:GCTRTRATAATGAGAAAGATTCTGC	52	60	714
ITS	F:GGAAGKARAAGTCGTAACAAGG<br>R:GTTTCTTTTCCTCCGCTTA	53	50	691

Supplementary Table S4 Information on the primers and amplification programs.

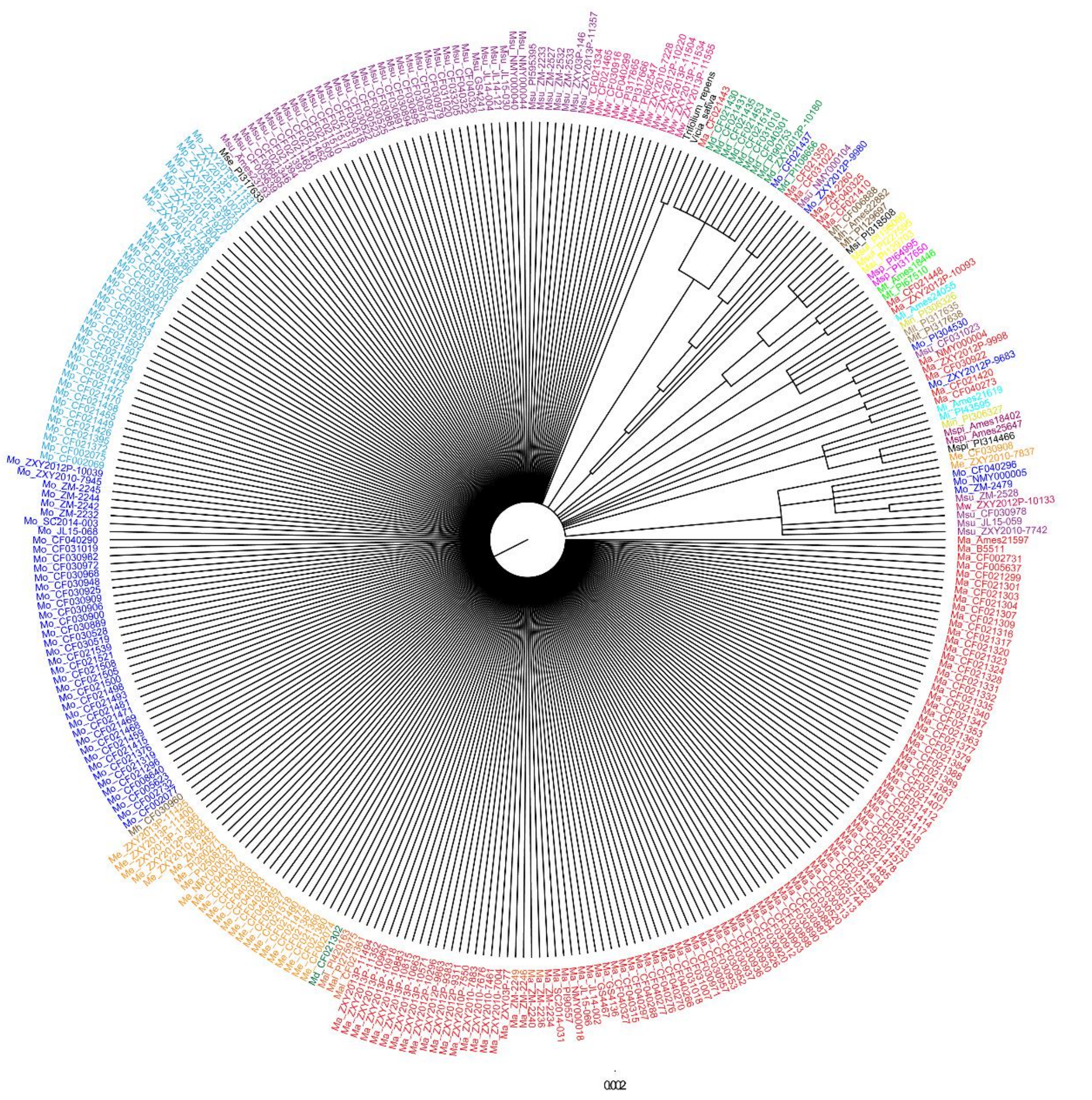

Supplementary Figure S1 Bayesian tree of 18 species in *Melilotus* with branch lengths, based on *mat*K sequences. The abbreviations represent 18 species: Ma—*M. albus*, Mal—*M. altissimus*, Md—*M. dentatus*, Me—*M. elegans*, Mh—*M. hirsutus*, Mi—*M. indicus,* Min—*M. infestus,* Mit—*M. italicus*, Mo—*M. officinalis*, Mp—*M. polonicus*, Mse—*M. segetalis*, Msi—*M. siculus*, Ms—*M. speciosus*, Mpi—*M. spicatus*, Msu—*M. suaveolens*, Msul—*M. sulcatus*, Mt—*M. tauricus*, and Mw—*M. wolgicus.* See Supplement Table S3 for accession numbers.

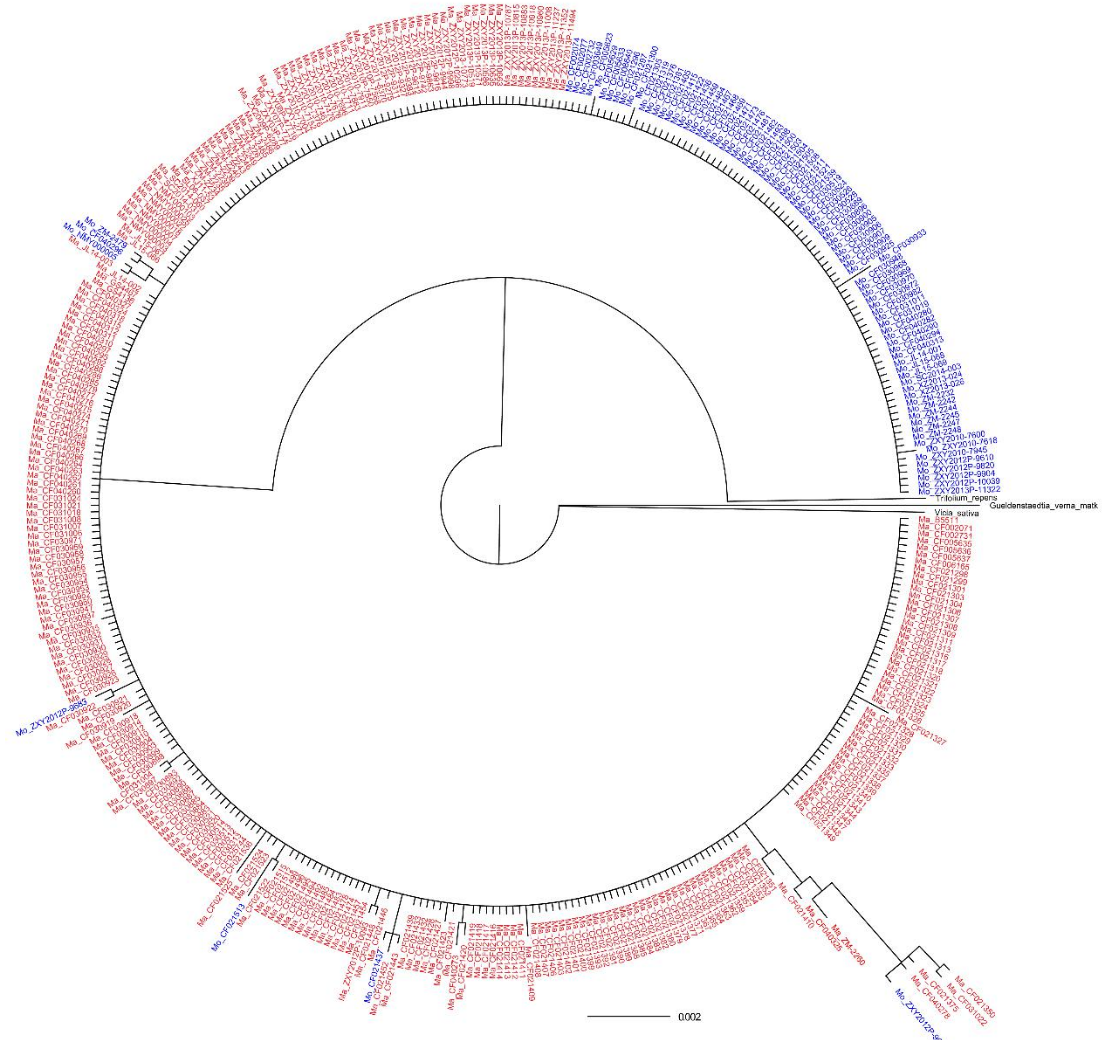

Supplementary Figure S2 Bayesian tree of *M. albus* and *M. officinalis* with branch lengths, based on *mat*K sequences. Ma—*M. albus* and Mo—*M. officinalis.* See Supplement Table S3 for accession numbers.
